# Absolute Configuration and Chiroptical Properties of Flexible Drug Avapritinib

**DOI:** 10.3390/ph18060833

**Published:** 2025-06-02

**Authors:** Ya-Dong Yang, Chen Zhao, Liang-Peng Li, Yi-Xin Lv, Bei-Bei Yang, Xin Li, Ru Wang, Li Li

**Affiliations:** State Key Laboratory of Digestive Health, Beijing Key Laboratory of Active Substances Discovery and Druggability Evaluation, Institute of Materia Medica, Chinese Academy of Medical Sciences & Peking Union Medical College, Beijing 100050, China; yangyadong@imm.ac.cn (Y.-D.Y.); zhaochen@imm.ac.cn (C.Z.); liliangpeng@imm.ac.cn (L.-P.L.); lvyixin@imm.ac.cn (Y.-X.L.); yangbetty@imm.ac.cn (B.-B.Y.); x_lixincc@163.com (X.L.); wangru@imm.ac.cn (R.W.)

**Keywords:** avapritinib, chiral resolution, flexible molecules, absolute configuration, chiroptical spectroscopy

## Abstract

**Background/Objective**: Avapritinib is an orally bioavailable tyrosine kinase inhibitor and was approved by the FDA in 2020 for gastrointestinal stromal tumor treatments. Although avapritinib is known to be chiral, its stereochemistry was initially established randomly. This study aims to develop a definitive method for determining avapritinib’s absolute configuration and propose a universal methodology for stereochemical characterization of flexible chiral drugs. **Methods**: The absolute configuration of avapritinib was determined through an integrated approach combining chiral resolution, chiroptical spectroscopy and synthetic validation. Enantiomeric separation was achieved via chiral liquid chromatography, followed by comprehensive chiroptical characterization including electronic circular dichroism (ECD), specific optical rotation and optical rotatory dispersion. Conformational analysis and density functional theory (DFT) calculations correlated experimental spectra with theoretical predictions, facilitating definitive configurational assignment. The stereochemical determination were further verified through ECD derivatization and chemical synthesis. Finally, the enantiomers’ kinase inhibition profiles against c-KIT D816V were quantitatively assessed. **Results**: Two enantiomers of avapritinib were resolved via chiral HPLC and a Chiralpak IG column. Through combined experimental ECD spectra and time-dependent DFT calculations employing the core extraction method, the *levo*-isomer was unambiguously determined as *S* configuration. This stereochemical assignment was confirmed by *p*-cyanobenzaldehyde derivatization and de novo synthesis. Biological evaluation revealed (*S*)-(−)-avapritinib exhibited superior c-KIT D816V inhibitory activity compared to its (*R*)-(+)-counterpart, a finding corroborated by molecular docking studies elucidating their differential target interactions. **Conclusions**: This study advances avapritinib stereochemical understanding and establishes a definitive protocol for its absolute configuration assignment, serving as a paradigm for flexible chiral drug characterization.

## 1. Introduction

The determination of absolute configuration constitutes a critical step in chiral drug characterization. Three principal techniques, X-ray crystallography [1], nuclear magnetic resonance (NMR) spectroscopy [2] and chiroptical spectroscopy [3], are commonly used to establish the absolute configuration of drugs. Chiroptical spectroscopic methods, such as electronic circular dichroism (ECD), vibrational circular dichroism (VCD), specific optical rotation (SOR) and optical rotatory dispersion (ORD), enable precise absolute configuration analysis when coupled with quantum chemical calculations [4,5,6]. Advancements in chiroptical spectroscopic techniques have facilitated absolute configuration determination for numerous chiral molecules.

Nevertheless, configurational analysis of conformationally flexible chiral molecules poses distinct analytical hurdles: X-ray crystallography necessitates diffraction-quality single crystals, a requirement particularly challenging for flexible systems owing to their diminished crystalline stability [7]. NMR spectroscopic approaches necessitate the presence of structurally appropriate derivatization sites within the chiral molecular framework [8]. Chiroptical methods, such as ORD, exhibit pronounced sensitivity to conformational dynamics in flexible systems [9,10]. These inherent technical constraints collectively contribute to the complexity of achieving unambiguous configurational assignment for flexible chiral compounds.

**Figure 1:**
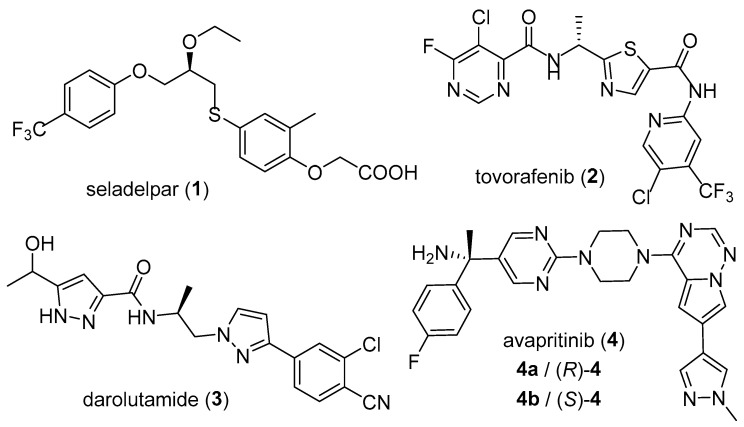
Representative flexible chiral drugs approved by the FDA.

Chirality represents a fundamental characteristic of both pharmaceutical compounds and natural products. For chiral drugs, the difference in stereochemical configuration might lead to completely different biological behaviors, including different pharmacodynamics, pharmacokinetics and toxicology parameters [11,12]. Notably, conformationally flexible scaffolds account for a substantial portion of commercial chiral drugs. In the last decade, some flexible chiral drugs with excellent pharmacological activities, such as seladelpar (**1**, Figure 1) [13], tovorafenib (**2**) [14], darolutamide (**3**) [15] and avapritinib (**4**) [16], have received FDA approval.

Avapritinib (**4**, Figure 1, BLU-285), a selective orally bioavailable tyrosine kinase inhibitor, demonstrates potent activity against mutant KIT and the KIT-D816V mutation of platelet-derived growth factor receptor alpha (PDGFRA) [17,18]. It was approved by the FDA for the treatment of PDGFRA exon 18 mutant gastrointestinal stromal tumors (GISTs) in 2020 and patients have demonstrated clinical benefit from its application [6,19]. Moreover, clinical trials of avapritinib for the treatment of indolent systemic mastocytosis and other diseases are underway [20]. Structurally, avapritinib is a linear flexible molecule with a chiral center on the rotatable chain. According to the initial patent applied by Blueprint company, the absolute configuration of avapritinib was originally randomly assigned [21]. To our knowledge, no systematic study has reported either the chiroptical characteristics or a validated protocol for its absolute configuration determination.

The aim of this study is to establish a conclusive approach for avapritinib’s absolute configuration determination while developing a generalized strategy for stereochemical analysis of conformationally flexible chiral drugs. The two enantiomers of avapritinib were first isolated using chiral liquid chromatography. Comprehensive chiroptical characterization was performed, including ECD, SOR, ORD and VCD analyses. Additionally, conformational analysis and DFT calculations were used to interpret the chiroptical behaviors. By comparing the calculated results with the experimental spectra, the absolute configurations of the two isomers were unambiguously assigned. In addition, the ECD derivatization method and de novo synthesis were used to confirm the configuration assignment. Differential kinase inhibition profiles of the enantiomers against c-KIT D816V were quantitatively assessed. This work enriched the stereochemical knowledge of avapritinib and also provided a feasible and reliable methodology to assign the absolute configuration of flexible chiral drugs.

## 2. Results

### 2.1. Chiral Resolution of Rac-***4***

Chromatographic techniques have emerged as the predominant methodology for enantiomeric separation. Major chromatographic modalities include gas chromatography (GC) [22], high performance liquid chromatography (HPLC) [23], supercritical fluid chromatography (SFC) [24] and capillary electrophoresis (CE) [25], each demonstrating unique enantioselectivity.

**Figure 2:**
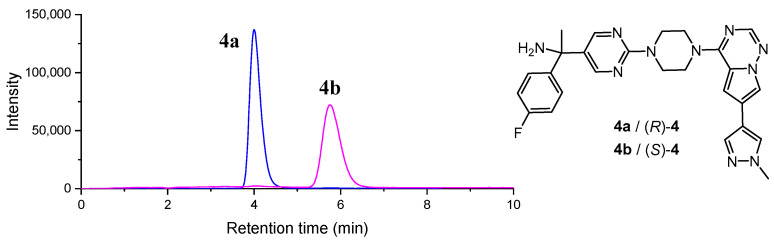
Chiral HPLC chromatograms of **4a** and **4b**. Retention times of **4a** and **4b**: **4a** (t_R_ = 4.01 min), **4b** (t_R_ = 5.76 min).

Previous studies employed SFC for avapritinib racemate analysis [21]. The chiral HPLC separation parameters were systematically optimized to achieve baseline resolution of enantiomers. Systematic method development involved screening multiple chiral stationary phases (Chiralpak AS, OJ, etc.) with various mobile phase combinations (isopropanol/n-hexane, ethanol/n-hexane). Optimal separation was achieved using a Chiralpak IG column (150 × 4.6 mm, 5 μm) under isocratic elution conditions with a mobile phase consisting of dichloromethane (CH_2_Cl_2_)/methanol (MeOH) (60:40, *v*/*v*) containing 0.1% diethylamine as an additive, yielding baseline resolution (Rs = 2.8) of enantiomers **4a** and **4b** (Figure 2, Table S1).

### 2.2. Experimental Chiral Spectroscopy of ***4a*** and ***4b***

#### 2.2.1. ECD Spectra

ECD spectroscopy serves as a robust tool for elucidating the absolute configuration of chiral compounds exhibiting UV–Vis absorption. Generally, this technique enables rapid and reliable stereochemical assignment through either empirical correlation with reference spectra or quantum-chemical calculations [26]. The Cotton effects (CEs) in the ECD spectra depend on the UV chromophores affected by surrounding chiral centers.

**Figure 3:**
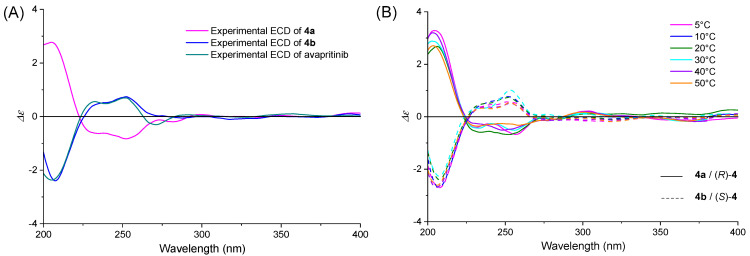
Experimental ECD spectra of **4a** and **4b** (**A**) in ACN; (**B**) at different temperatures, **4a**: solid line, **4b**: dash line.

The chromophores in avapritinib are *p*-fluorophenyl, substituted pyrimidinyl and 6-(1H-pyrazol-4-yl)pyrrolo[2,1-*f*][1,2,4]triazinyl groups, which all have strong absorption in the UV region. The conformational flexibility of **4** results in opposing CE contributions from different conformers, leading to strong UV absorption but attenuated ECD signals. Therefore, it is not easy to collect the ECD spectra of its enantiomers. The enantiomers exhibited mirror-image ECD spectra in acetonitrile (ACN), with **4a** displayed a positive CE at 210 nm and two negative CEs at 231.5 nm and 253.5 nm (Figure 3A). Meanwhile, **4b** showed the opposite curve and the same as the commercial avapritinib, indicating that they had the same configuration. Solvent-dependent spectral variations of **4a** and **4b** were observed, with methanol and aqueous hydrochloride solutions inducing minor CEs intensity modifications (Figure S1), attributable to hydrogen-bonding interactions.

Since the ECD spectrum is sensitive to changes in the conformational equilibrium mixture, variable-temperature ECD (VT-ECD) tests of **4a** and **4b** were carried out. As shown in Figure 3B, there was no obvious change when the temperature increased from 5 to 50 °C, indicating that the distribution of conformations remained almost unchanged over this temperature range.

#### 2.2.2. ORD

SOR constitutes an intrinsic chiral property of chiral compounds, while ORD characterizes its wavelength dependence [27]. Notably, ORD and ECD spectra exhibit mathematical interconversion within the UV–Vis region through Kramers–Kronig transformations [26]. For non-UV-absorbing chiral molecules, ORD can serve as an important tool for determining absolute configurations.

**Figure 4:**
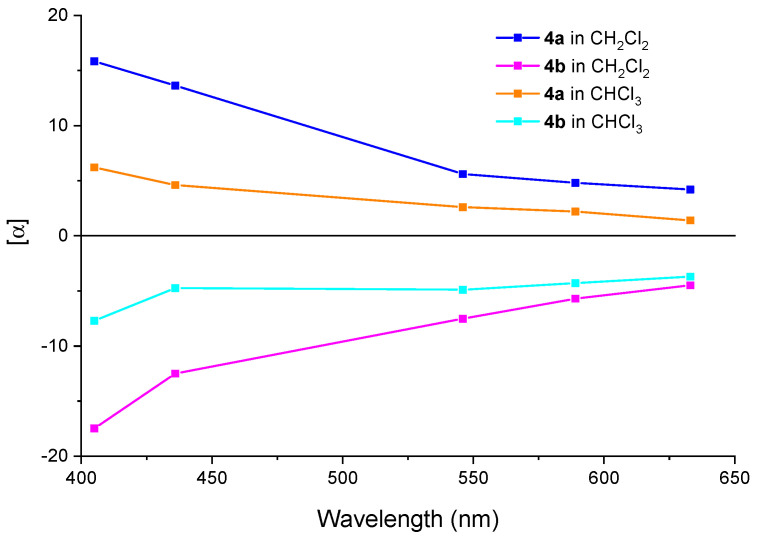
Experimental ORD of **4a** and **4b** in CH_2_Cl_2_ and CHCl_3_.

Initial ORD measurements of enantiomers **4a** and **4b** were conducted in dichloromethane (CH_2_Cl_2_). The first-eluting enantiomer **4a** was dextrorotatory, while the second elution **4b** showed levorotation (Figure 4).

The ORD of **4a** and **4b** were in an antipodal relationship, and **4a** gave a flat curve that increased from 405 nm to 633 nm. Complementary ORD measurements in chloroform (CHCl_3_) revealed solvent-induced spectral shifts, though SOR determination at 365 nm was precluded by detector limitations of the dark mode. It is shown that the solvent affected the values rather than the sign of SORs.

#### 2.2.3. VCD Spectra

VCD spectroscopy quantifies the differential absorption of left- and right-circularly polarized infrared radiation by chiral molecules, delivering direct stereochemical information through characteristic vibrational transitions. As a powerful technique for absolute configuration determination, VCD offers complementary structural insights to ECD, particularly for molecules lacking UV chromophores. Key experimental limitations in VCD measurements, particularly low sample solubility and poor signal-to-noise ratios, significantly constrain its applications [28].

In fact, we encountered these difficulties when collecting VCD signals of **4a** and **4b**. Their VCD signals in dimethyl sulfoxide-*d_6_* and CDCl_3_ were too weak to detect, and we did not obtain a satisfactory full range spectrum after multiple attempts. Conformational flexibility induces spectral cancellation effects, where opposing VCD signals from different rotamers yield net intensity attenuation [29]. Structural complexity further diminishes spectral resolution, obscuring diagnostically critical differential signals. Consequently, VCD proves suboptimal for absolute configuration determination in flexible, extended-chain molecules like avapritinib. In addition, the occurrence of solute aggregation in the solvent at the relatively large concentration used for VCD measurements could result in failure [30].

### 2.3. Absolute Configuration Assignment of ***4a*** and ***4b***

#### 2.3.1. Conformational Analysis of **4**

**Figure 5:**
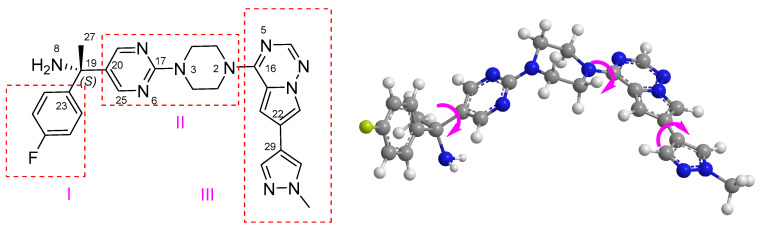
Two-dimensional and three-dimensional structures of (*S*)−**4**.

A systematic conformational search of **4** was performed for the (*S*)-**4** isomer to give a total of 96 conformations. Structural analysis identified three distinct molecular fragments, with fragment II constituting the core scaffold (Figure 5). Conformational diversity arises from rotational freedom about C19-C20, C17-N3, and N2-C16 bonds, generating distinct spatial arrangements. Thus, these 96 conformations could be divided into eight groups.

First, two fundamental groups are divided according to the conformation of piperazine ring in fragment II, as shown in Table S2. Fragments I and III could stay on the same side (cis-) or different side (trans-) of fragment II. Second, due to the rotation of the C19−C20 bond, the relative position between fragments I and II may change, and the dihedral angle C27−C19−C20−C25 value was close to 180° (methyl coplanar) or within the range from 50° to 100° (methyl non-coplanar). Then, the fluorine atom on fragment I and the pyrazole ring of fragment III move in the same direction (*Z*-) and different directions (*E*-). Finally, the terminal methyl group on the pyrazole ring may be close to or far from the skeleton due to the rotation of the C22−C29 bond. The six conformations within the same group had similar or complementary dihedral angles C13−C15−C19−C27 (D1), C30−C23−C19−N8 (D2) and C26−C20−C19−C27 (D3). Thus, the relative positions of fragments I, II and III might play an important role in the chiroptical properties.

Absolute configuration determination exhibited notable sensitivity to computational parameters, influencing both conformational distributions and subsequent ECD spectral simulations [10,27]. To address this, we systematically evaluated three distinct functional/basis set combinations for the conformational distribution analysis, with explicit solvent effects incorporated via the SMD continuum solvation model [26,31].

To calculate the Boltzmann populations of these conformations, three computational protocols were employed: M1: SMD/ACN/B3LYP/6-31G(d,p); M2: SMD/CH_2_Cl_2_/B3LYP/6-31G(d,p); M3: SMD/CH_2_Cl_2_/B3LYP/6-31++G(d,p) (Figure S2). Due to the molecular flexibility and their similar Gibbs free energies, the Boltzmann populations for most conformers ranged within 2%. However, it is obvious that different combinations of calculation parameters exerted substantial influence on the Boltzmann distribution.

#### 2.3.2. ECD Simulation

**Figure 6:**
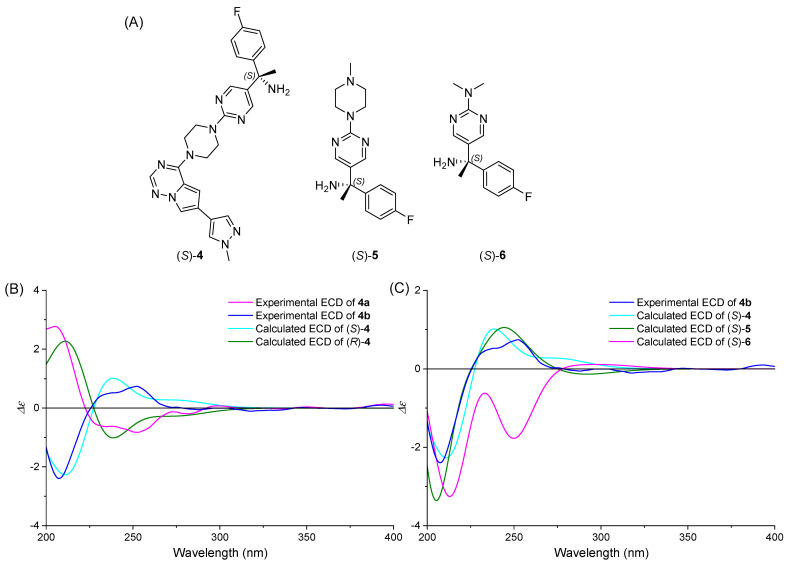
(**A**) Structures of compounds (*S*)-**4**-**6**. (**B**) Comparison of the theoretical ECD spectra of (*R*)-**4** and (*S*)-**4** with the experimental spectra of **4a** and **4b** in ACN, SMD/ACN/ωB97XD/TZVP// ωB97XD/TZVP, σ = 0.40 eV. (**C**) Comparison of the calculated ECD of (*S*)-**4**-**6** with the experimental ECD of **4b** in ACN, SMD/ACN/B3LYP/6-31G(d,p)//B3LYP/6-31G(d,p), σ = 0.40 eV.

Following conformational analysis, ECD calculations of (*S*)-**4** (Figure 6A) were performed using time-dependent density functional theory (TDDFT). Different conformers showed completely different ECD curves, indicating that the relative position of fragments I, II and III had a great influence. The overall ECD of (*S*)-**4** isomer was obtained by summarizing the spectra of all conformers based on Boltzmann populations. As shown in Figure 6B, the predicted ECD curve of (*S*)-**4** has a positive band at 211 nm as well as two negative bands at 235 nm and 257 nm, exhibiting 0.873 similarity index with experimental ECD of **4b**. Therefore, we preliminarily determined that the second eluted enantiomer **4b** had an “*S*” configuration.

In the three-dimensional structure of (*S*)-**4**, the rotation of the N2-C16 bond greatly increased the number of conformations. Despite the distal location of fragment III relative to the chiral center, its conformational variations induced significant spectral divergence, complicating ECD analysis. Thus, we tried the method of extracting the core structure [31] to simplify the calculation. When the structure of (*S*)-**4** was truncated as (*S*)-**5** and (*S*)-**6**, the similarity factors of the relative theoretical spectra and the experimental ECD curve of **4b** were 0.968 and 0.659, respectively (Figure 6C). Obviously, rationally simplifying the structure of flexible molecules can improve the analysis efficiency, but excessive simplification results in decreased similarity of the spectra.

As to the simplified structure (*S*)-**5**, only 17 conformations were obtained, which greatly reduced the difficulty of analysis and calculation. Minor spectral variations were observed across computational methods: B3LYP/6-31G(d,p), Cam-B3LYP/6-311G(d,p) and ωB97XD/TZVP (Figure S3). All these theoretical results were similar to the experimental results with a high similarity factor.

#### 2.3.3. SOR and ORD Simulation

SOR at the sodium D line (589.3 nm) represents the most widely employed chiroptical parameter for characterizing optically active compounds and can be theoretically predicted with satisfactory accuracy using modern quantum chemical methods [32]. However, it is still difficult to calculate the SOR value for flexible molecules owing to their conformational diversity [33]. For (*S*)-**4**, the SOR and ORD were calculated under the SMD or PCM solvation model in CH_2_Cl_2_. Unfortunately, after attempting various parameter combinations, we were unable to obtain stable and consistent ORD data. Minor variations in computational parameters or conformational populations induced substantial deviations in the calculated values, causing the sign of the overall result to randomly change. The application of machine learning of the relationship between SOR and conformation might be an alternative way to solve this problem in the future.

### 2.4. Confirmation of Absolute Configuration Assignment

#### 2.4.1. ECD Derivatization Method

**Scheme 1:**
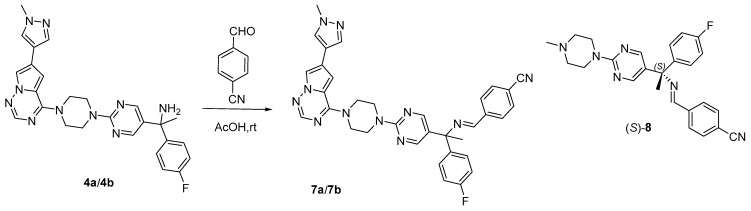
Derivatization method of **4** and the simplified structure (*S*)-**8**.

Enantiopure **4** was subjected to chemical derivatization, and the two isomers **4a** and **4b** were transformed into corresponding Schiff’s bases **7a** and **7b** via condensation with *p*-cyanobenzaldehyde (Scheme 1).

**Figure 7:**
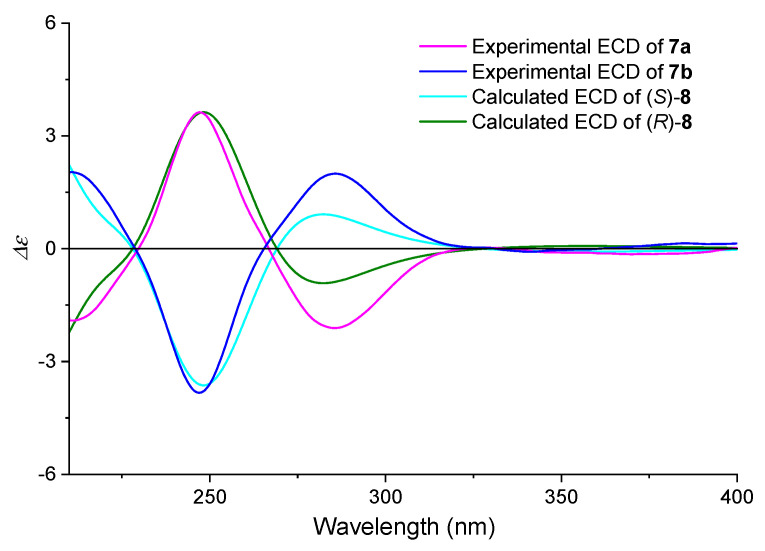
Comparison of the calculated and experimental ECD spectra of compound **7** in ACN. (SMD/ACN/ωB97XD/TZVP//ωB97XD/TZVP, σ = 0.40 eV).

As shown in Figure 7, the two derivatives showed mirror signals with the typical characteristics of exciton-coupling circular dichroism. Characteristic bisignate signals appeared at 247 nm and 284.5 nm, which could be ascribed to the exciton coupling between pyrimidinyl group and aryl Schiff bases.

To confirm the feasibility of the method in extracting the core structure, we also simplified the structure of derivative **7** and removed fragment III to yield compound **8**. The computationally simulated ECD spectrum of (*S*)-**8** exhibited excellent agreement with the experimental curve of **7b**. Thus, by comparing with the predicted ECD spectra of (*S*)/(*R*)-**8**, derivative **7b** was assigned an *S* configuration. Furthermore, this result verified that the method of extracting core structure and removing the fragment III of **4** to simplify the ECD calculation was reasonable.

#### 2.4.2. Chemical Correlation Method

**Scheme 2:**
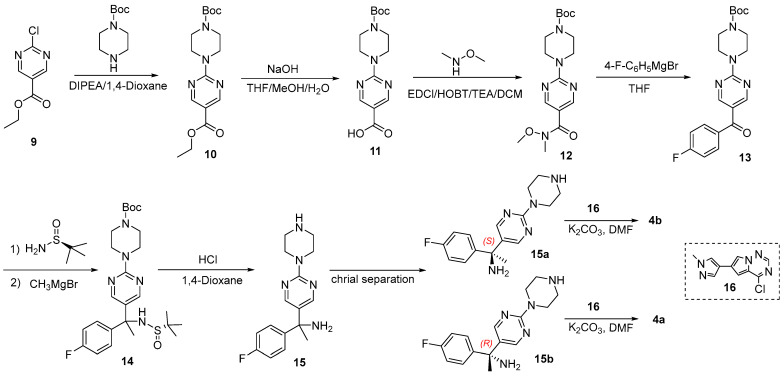
Synthesis route of **4** based on the related patent [21].

Following the patented synthetic route (Scheme 2) [21], two stereoisomers of **4** were successfully prepared.

The synthesis commenced with nucleophilic substitution of compound **9**, followed by Grignard reaction of intermediate **12** to afford compound **13**. (*S*)-2-Methylpropane-2-sulfinamide was selected to introduce the amino group. Racemic **15** was obtained following the hydrolysis reaction of **14**.

**Figure 8:**
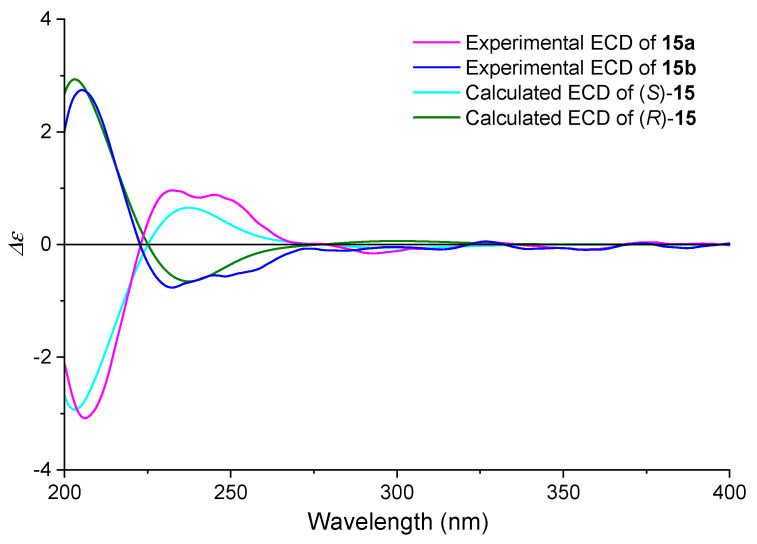
Comparison of the calculated and experimental ECD spectra of compound **15** in ACN. (SMD/ACN/wb97xd/TZVP//wb97xd/TZVP, σ = 0.40 eV).

Enantiomeric resolution was achieved using chiral HPLC (CHIRALPAK ID column), with the elution order defining the designation of **15a** (first eluting) and **15b** (second eluting), and their ECD curves were collected. Comparative analysis of experimental and calculated ECD spectra (Figure 8) unambiguously assigned the *S* configuration to **15a** and *R* configuration to **15b**. The near-identical ECD profiles of **15a** and **4b** demonstrated minimal chiroptical perturbation upon fragment III excision, validating the core structure extraction strategy.

Additionally, the ORD spectra of **15a** and **15b** were tested in CH_2_Cl_2_ (Figure S4), and the values were approximately 20 times that of **4**. This phenomenon likely stems from the relative larger chiral center density in **15** compared to the full molecule **4**. The absence or presence of fragment III had a great influence on SOR, which was a reason why the configuration assignment of **4** failed using the ORD method.

Once the absolute configuration of **15a** and **15b** was determined, the synthetic route was investigated to obtain the object compound **4**. Theoretically, **4b** was obtained from intermediates **15a** and **4a** from **15b**. The ECD spectral correlation provided conclusive evidence supporting our configurational assignment.

**Scheme 3:**
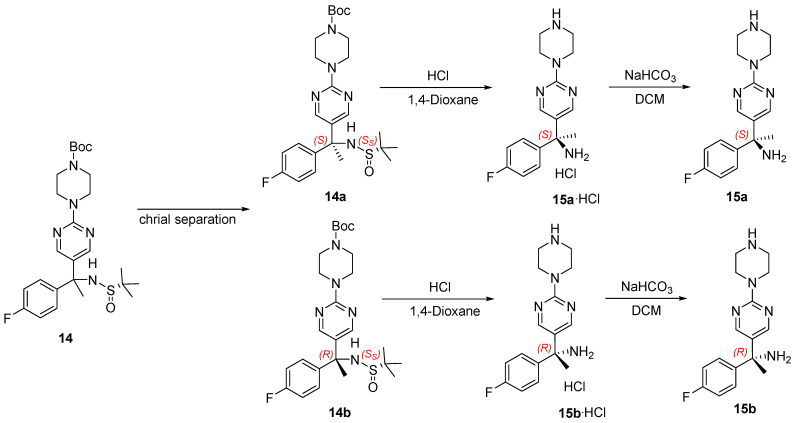
Resolution of epimers **14a** and **14b**.

Additionally, two isomers of **14** were separated (Scheme 3), and epimers **14a** and **14b** were obtained by Chiralpak AS column resolution. The experimental ECD spectra of **14a** and **14b** showed excellent agreement with the calculated ECD curves for (*S*,*S_S_*)-**14** and (*R*,*S_S_*)-**14**, respectively (Figure S5).

After hydrolysis with hydrochloric acid, the hydrochloride form of compound **15** was obtained. The calculated and experimental ECD patterns exhibited high similarity, and the salt obtained from **14a** could be judged to have an *S* configuration (Figure S6). The ECD experimental curves obtained after alkalization also correspond to **15a** and **15b** obtained by the previous resolution, which further demonstrates the configuration determined, which was **15a** in the *S* configuration and **15b** in the *R* configuration.

### 2.5. Effect of Configuration on Biological Activity

With the two enantiomers **4a** and **4b** in hand, their inhibitory activity against kinase C-KIT (D816V) was evaluated, using amuvatinib [34] as the positive control (Table 1). Stereochemical configuration exerted significant influence on inhibitory potency, with nearly 5-fold activity differential observed between enantiomers. Enzymatic profiling confirmed **4b** as the eutomer (IC_50_ = 0.59 ± 0.10 nM), demonstrating comparable efficacy to clinical-grade avapritinib (IC_50_ = 0.5 ± 0.1 nM) [35].

**Figure 9:**
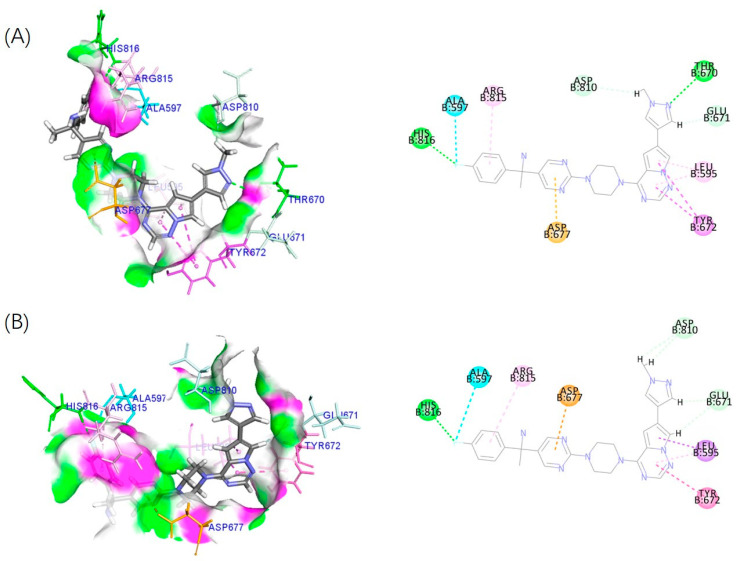
Predicted binding modes of (**A**) (*S*)-**4** and (**B**) (*R*)-**4** with the C-KIT mutant (PDB code: 3G0F). Conventional hydrogen bonds are shown as green dashed lines, π-σ interactions as deep purple dashed lines, π-π stacked interactions as purple dashed lines, π-anion interactions as orange dashed lines, π-alkyl interactions as pink dashed lines and the residues that has van der Waals interactions with this molecule are in light green color, the interactions between halogen and ALA597 are shown in blue lines.

Molecular docking simulations elucidated the binding model between the enantiomers of **4** and the C-KIT mutant (Figure 9).

The results indicated that fragments I and II of two enantiomers had similar interactions with the surrounding amino acid residues. The molecular interactions can be characterized as follows: a π-anion interaction occurs between the carboxylate group of ASP677 within the binding pocket and the electron-deficient pyrimidine ring system in fragment II for both two enantiomers. Simultaneously, a π-alkyl interaction forms between the guanidinium group of ARG815 and the aromatic system of the benzene ring in fragment I, while a hydrogen bond is established between the imidazole nitrogen of HIS816 and the fluorine atom in fragment I. Hydrophobic stabilization is provided through van der Waals contacts involving the side chains of LEU595 and TYR672 with the pyrrolo[2,1-*f*][1,2,4]triazine moiety in fragment III. However, the pyrazole in fragment III of **4b**/(*S*)-**4** could generate additional hydrogen bond interactions with key amino acid residue Thr670, which was more advantageous than **4a**/(*R*)-**4**.

## 3. Conclusions

In this study, a novel integrated strategy for determining the absolute configuration of flexible chiral molecules is presented, using avapritinib (**4**) enantiomers as a model system. Through comprehensive chiroptical characterization, we unambiguously established the (*R*)-configuration for dextral-isomer **4a** and (*S*)-configuration for *levo*-isomer **4b**. We established an optimized analytical protocol that achieves a balance between analytical accuracy and operational efficiency through strategic structural simplification. This methodology demonstrates broad applicability for characterizing diverse conformationally flexible compounds. Our multi-method validation approach, combining derivatization, chemical synthesis and advanced spectroscopic techniques, consistently confirmed the (*S*)-configuration of marked avapritinib. The biological relevance was demonstrated through kinase inhibition assays, revealing the (*S*)-enantiomer’s enhanced inhibitory activity against C-KIT (D816V) compared to its counterpart.

This work presents the first systematic comparison of chiroptical techniques for analyzing conformationally flexible avapritinib, addressing critical knowledge gaps in this field. The developed structure-simplification approach demonstrates significant improvements in analytical efficiency while rigorously maintaining experimental reliability standards. Furthermore, the study proposes a generalizable framework for unambiguous absolute configuration determination of pharmaceutically relevant flexible chiral compounds, offering broad applicability in drug discovery and development.

## 4. Materials and Methods

### 4.1. General Information

Racemic avapritinib and marketed chiral avapritinib were purchased from Hanxiang Biochempartner and Bidepharm, respectively. Chromatographic resolution was carried out at room temperature on a Shimadzu HPLC2010A system. Chiralpak OD/AS/OJ/IG/ID chiral columns were used to explore suitable conditions. Chromatogram acquisitions and elaborations were performed using the LC solution 1.0 software. The SOR and ORD spectra were collected on Rudolph Autopol IV (Rudolph, NJ, USA). The analysis was conducted using a 0.5 dm cell, chloroform (CHCl_3_) or dichloromethane (CH_2_Cl_2_) as solvents. Reported optical rotation values represent triplicate measurements at six wavelengths 365, 405, 436, 546, 589 and 633 nm. ECD spectra were recorded using a Jasco J-815 spectrometer (Jasco, Tokyo, Japan) at room temperature in spectroscopic grade solvents. All spectra were recorded with a path length of 0.1 cm, using a scanning speed of 100 nm min^−1^, a bandwidth of 1 nm and an accumulation of 2 scans. VT-ECD measurements were carried out attached to a PTC-423S temperature control device in the temperature range from 5 to 50 °C, using the measurement parameters listed above. VCD spectra were obtained using a Jasco FVS-6000 spectrometer (Jasco, Tokyo, Japan) at a resolution of 4 cm^−1^ in the range of 2000–850 cm^−1^ in spectroscopic grade DMSO-d_6_ and CDCl_3_.

As for chemical synthesis, all solvents and reagents were purchased from commercial suppliers, such as Innochem and Beijing Tong Guang Fine Chemicals Company and directly used without further purification. The silica gel column was 200–300 mesh (Qingdao Ocean Chemical Plant), and the TLC silica gel column was GF254 silica gel (Qingdao Ocean Chemical Plant). NMR spectra were recorded in CDCl_3_, MeOD or DMSO-d_6_ with a JOEL JNMR-ECZS 400 MHz spectrometer (JOEL, Tokyo, Japan) or Bruker Avance-III 400 NMR spectrometer (Bruker, Karlsruhe, Germany) using TMS as internal standard at ambient temperature. The chemical shifts (δ) are expressed in parts per million (ppm) downfield, and coupling constant (J) values are expressed in hertz. MS experiments were conducted on a Thermo Extractive plus Orbitrap mass spectrometer (Thermo Fisher, Waltham, MA, USA) with an electrospray ionization (ESI) source.

### 4.2. Computational Section

The MOE software (2015.10) was used to search for conformations in the molecular force field of MMFF94, and all conformations in the energy range of 10 kcal/mol were regarded as initial conformations [36]. The DFT calculations were carried out with Gaussian16 RevB.01 [37]. The Boltzmann distributions of the compounds at 298.15 K and 1 atm were calculated according to their Gibbs free energy calculations. The SOR calculation step was run under the static limit of B3LYP/6-31G(d,p), ωB97XD/6-311G(d,p) or ωB97XD/TZVP level. The SMD model was applied as the solvation model, and SpecDis 1.71 software [38,39] was used to obtain the averaged ECD and SOR calculated according to Boltzmann distribution.

### 4.3. Chemical Synthesis

#### 4.3.1. Synthesis of Derivatives **7a**/**7b**

Each of **4a** and **4b** (1 mg, 2 mmol) was dissolved in acetic acid and 4-cyanobenzaldehyde 0.52 mg (4 mmol) was subsequently added. The two reactions were carried out at room temperature for two days. The reaction mixture was concentrated for the ECD test.

#### 4.3.2. Synthesis of **4a** and **4b**

Synthesis of **10**: Ethyl 2-chloropyrimidine-5-carboxylate (1.9 g, 10 mmol) was added to a solution of *tert*-butyl pipierazine-1-carboxylate (**9**, 1.9 g, 10 mmol) and diisopropylethylamine (4 mL) in dioxane, and the reaction mixture was stirred at room temperature (RT) for 3 h. LC-MS results showed that the reaction was completed. The reaction was concentrated to afford white crude compound **10** (3.6 g), which was directly used in the next step without the further purification. m.p. 123–125 °C. ^1^H NMR (400 MHz, DMSO-*d_6_*): δ 8.80 (s, 2H), 4.27 (q, J = 7.1 Hz, 2H), 3.87–3.81 (m, 4H), 3.42 (t, J = 5.3 Hz, 4H), 1.42 (s, 9H), 1.29 (t, J = 7.1 Hz, 3H) ppm. HRMS (ESI): m/z [M + H]^+^ Calcd. for C_16_H_24_N_4_O_4_: 337.1870; found 337.1862.

Synthesis of **11**: Compound **10** (3.6 g, 10 mmol) was dissolved in 60 mL THF/MeOH/water, NaOH (0.84 g, 21 mmol) was added, and the reaction was stirred at 70 °C for 3 h. LC-MS results indicated that the reaction was completed. The reaction mixture was cooled to RT and then acidified to pH 5 with 1 M hydrochloric acid. The precipitated solid was filtered by suction filtration. The solid was collected and dried to give the title compound **11** (3.0 g) as a white solid, with a yield of 97.4%. m.p. 157–159 °C. ^1^H NMR (400 MHz, CDCl_3_): δ δ 8.86 (s, 2H), 3.93 (d, J = 5.7 Hz, 4H), 3.52 (t, J = 5.3 Hz, 4H), 1.49 (t, J = 2.2 Hz, 9H) ppm. ESI-HRMS: m/z [M + H]^+^ Calcd. for C_14_H_20_N_4_O_4_: 309.1557; found 309.1552.

Synthesis of **12**: Compound **11** (3.1 g, 10 mmol), EDCI (2.9 g, 15 mmol) and HOBT (1.6 g, 12 mmol) were dissolved in 60 mL dichloromethane, and followed by 5 mL triethylamine (40 mmol). The mixture was stirred at RT for 1 h, and then N,O-dimethylhydroxylamine (1.2 g, 12 mmol) was added. The reaction was stirred for another 3 h. LC-MS results showed that the reaction was completed. The reaction mixture was washed with water (30 mL), and the organic layer was dried, filtered and concentrated. The residue was purified by silica gel chromatography (petroleum ether: ethyl acetate = 2:1) to give the compound **12** (1.8 g) as a white solid, with a yield of 52.6%. m.p. 130–132 °C. ^1^H NMR (400 MHz, CDCl_3_): δ 8.79 (d, J = 1.8 Hz, 2H), 3.88 (t, J = 5.3 Hz, 4H), 3.60 (s, 3H), 3.49 (t, J = 5.3 Hz, 4H), 3.34 (s, 3H), 1.47 (s, 9H) ppm. ESI-HRMS: m/z [M + H]^+^ Calcd. for C_16_H_25_N_5_O_4_: 352.1979; found 352.1999.

Synthesis of **13**: Compound **12** (3.0 g, 8.6 mmol) was dissolved in 30 mL of dry THF, and 10 mL of C_6_H_5_MgFBr was added at 0 °C under nitrogen. The mixture was stirred at RT for 3 h. LC-MS results demonstrated that the reaction was completed. Saturated sodium chloride (10 mL) was added to the reaction and extracted with ethyl acetate. The combined organic layers were dried over sodium sulfate, filtered and concentrated. The residue was purified by silica get chromatography (PE: EA = 3:1) to generate compound **12** (2.3 g) as a white solid, with a yield of 69.7%. m.p. 180–182 °C. ^1^H NMR (400 MHz, CDCl_3_): δ 8.81 (s, 2H), 7.81–7.76 (m, 2H), 7.19 (t, J = 8.4 Hz, 2H), 4.05–3.99 (m, 4H), 3.57 (t, J = 5.2 Hz, 4H), 1.50 (s, 9H) ppm. ESI-HRMS: m/z [M + H]^+^ Calcd. for C_20_H_23_FN_4_O_3_: 387.1827; found 387.1796.

Synthesis of **14**: (*S*)-2-Methylpropane-2-sulfinamide (1.8 g, 15 mmol), compound **13** (1.8 g, 5 mmol) and ethyl orthotitanate (2 mL, 10 mmol) were stirred in 30 mL of dry THF at 70 °C under nitrogen for 24 h. LC-MS results showed the reaction was completed. When the reaction was dropped to room temperature, 10 mL water was added. Then, the product was extracted with ethyl acetate. The combined organic layers were washed with water, dried over sodium sulfate, filtered and concentrated. Finally, 2.5 g of yellow intermediate was obtained. Then, the intermediate (2.45 g, 5 mmol) was taken up in 30 mL of dry THF and cooled to 0 °C. CH_3_MgBr (3 M solution in diethyl ether, 15 mL, 25 mmol) was added and the resulting mixture stirred at 0 °C for 45 min. Additional CH_3_MgBr (3 M solution in diethyl ether, 3 mL, 5 mmol) was added and stirred at 0 °C for 20 min. Saturated ammonium chloride was added, and the products were extracted into EtOAc. The combined organic layers were washed with water, dried over sodium sulfate, filtered and concentrated. Compound **14** (2.4 g) was obtained as a yellow solid, with a yield of 94.8%. m.p. 215–217 °C. ^1^H NMR (400 MHz, CDCl_3_): δ 8.26 (s, 1H), 8.21 (s, 1H), 7.30 (dt, J = 9.4, 4.7 Hz, 2H), 6.97 (q, J = 7.4 Hz, 2H), 3.75 (t, J = 5.1 Hz, 4H), 3.44 (t, J = 5.2 Hz, 4H), 1.99 (s, 3H), 1.43 (s, 9H), 1.18 (d, J = 6.8 Hz, 9H)) ppm. ESI-HRMS: m/z [M + H]^+^ Calcd. for C_25_H_36_FN_5_O_3_S: 506.2596; found 506.2627.

Synthesis of **15**: Compound **14** (2.4 g) was stirred in 4 M HCl in 1,4-dioxane (30 mL)/MeOH (30 mL) at room temperature for 2 h. The solvent was removed in vacuo, and the residue was triturated in EtOAc to generate 1.1 g of compound **15** as a yellow solid, with a yield of 76.8%. m.p. 111–113 °C. ^1^H NMR (400 MHz, CDCl_3_): δ 8.38 (d, J = 1.9 Hz, 2H), 7.43 (dd, J = 8.2, 4.5 Hz, 2H), 7.22 (t, J = 8.5 Hz, 2H), 4.11 (d, J = 5.4 Hz, 4H), 3.28 (d, J = 5.3 Hz, 4H), 2.08 (s, 3H) ppm. ESI-HRMS: m/z [M+H]^+^ Calcd. for C_16_H_20_FN_5_: 302.1776; found 302.1783. After chiral resolution of **15** (300 mg), enantiomers **15a** and **15b** were obtained. **15a**: ^1^H NMR (500 MHz, MeOD): δ 8.39 (s, 2H), 7.44 (ddd, J = 8.9, 4.9, 2.4 Hz, 2H), 7.23 (ddt, J = 8.7, 6.6, 2.6 Hz, 2H), 4.16–4.11 (m, 4H), 3.29 (dd, J = 6.0, 4.7 Hz, 4H), 2.09 (s, 3H) ppm. **15b**: ^1^H NMR (500 MHz, MeOD): δ 8.39 (s, 2H), 7.46–7.41 (m, 2H), 7.26–7.20 (m, 2H), 4.15–4.11 (m, 4H), 3.30–3.27 (m, 4H), 2.09 (s, 3H) ppm.

Synthesis of **4a**/**4b**: Compounds **15a** and **15b** (1.2 mg, 0.4 mmol) were dissolved in 10 mL EtOH respectively, 0.24 mL DIPEA was added, and then 4-chloro-6-(1-methyl-1H-pyrazol-4-yl)pyrrolo[2,1-*f*][1,2,4]triazine(1.0 mg, 0.4 mmol) was dissolved in 5 mL EtOH and dropped into the reaction solution at 0 °C. Subsequently, the reaction was moved to room temperature, and the completion of the reaction was detected after 5 h. LC-MS results showed that the reaction was completed. The reaction mixture was concentrated, and the residue was purified by silica get chromatography (CH_2_Cl_2_: EtOH = 20:1). Compounds (*S*)-**4** (1.1 mg) and (*R*)-**4** (1.3 mg) were obtained as white solids, the yields were 55% and 65%, respectively. **4a**: ^1^H NMR (500 MHz, CDCl_3_): δ 8.39 (s, 2H), 7.90 (s, 1H), 7.70 (dd, J = 2.2, 1.2 Hz, 2H), 7.56 (d, J = 0.8 Hz, 1H), 7.42–7.38 (m, 2H), 7.07–7.03 (m, 2H), 6.78 (d, J = 1.7 Hz, 1H), 4.17–4.14 (m, 4H), 4.05–4.01 (m, 4H), 3.95 (s, 3H), 1.96–1.91 (m, 3H) ppm. ESI-HRMS: m/z [M–NH_3_+H]^+^ Calcd. for C_26_H_27_FN_10_: 482.2211; found 482.2213. **4b**: ^1^H NMR (500 MHz, CDCl_3_): δ 8.39 (s, 2H), 7.90 (s, 1H), 7.70 (dd, J = 3.2, 1.2 Hz, 2H), 7.57–7.56 (m, 1H), 7.42–7.38 (m, 2H), 7.05 (t, J = 8.6 Hz, 2H), 6.78 (d, J = 1.7 Hz, 1H), 4.17–4.13 (m, 4H), 4.05–4.01 (m, 4H), 3.95 (s, 3H), 1.95 (s, 3H) ppm. ESI-HRMS: m/z [M–NH_3_+H]^+^ Calcd. for C_26_H_27_FN_10_: 482.2211; found 482.2213.

### 4.4. Docking Analysis

A molecular docking study was executed by applying Discovery Studio 2019. First, the X-ray crystal structure of the C-KIT mutant was retrieved from the Protein Data Bank (PDB code: 3G0F) and prepared for use. Then, the receptor grid was generated at the centroid of the original ligand and the grid box size was set to 12 Å. After ligands **4a** and **4b** were prepared and minimized, molecular docking was carried out using LibDock with the default settings.

### 4.5. Biological Activity Assays

The kinase activity was tested by ICE Bioscience Inc. Kinase C-KIT (816V) seeded in flat-bottom 384-well plates and treated with the tested compounds, followed by the addition of substrate/ATP solution at 0.4 mg/mL in one well after 10 min incubation. After treatment for at 25 °C for 30 min, the detection buffer was added and kept reacting for 60 min. Then, the results were determined using ELISA-BMG. The quantitative results are expressed as the mean values ± SD, calculated using GraphPad Prism 8.

## Figures and Tables

**Table 1:** The inhibitory activities of **4a** and **4b** against kinase C-KIT (D816V).

Compounds	IC_50_ (nM)
**4a** ((*R*)-**4**)	2.81 ± 0.32
**4b** ((*S*)-**4**)	0.59 ± 0.10
Amuvatinib	26.89 ± 1.51

## Data Availability

The original contributions presented in the study are included in the article and Supplementary Material, further inquiries can be directed to the corresponding author.

## Supplementary Materials

The following supporting information can be downloaded at: https://www.mdpi.com/article/10.3390/ph18060833/s1, Table S1: chiral HPLC conditions of **4** and **15**; Table S2: Conformational grouping of (*S*)-**4**; Figure S1: Experimental ECD spectra of **4a** and **4b** in methanol and water; Figure S2: Boltzmann distribution of (*S*)-**4** obtained using three calculation methods; Figure S3: Comparison of the experimental ECD spectrum of **4b** with the calculated ECD spectra of (*S*)-**5** in ACN using different methods; Figure S4: ORD curves of **15a** and **15b** in CH_2_Cl_2_; Figure S5: Comparison of the experimental ECD spectra of **14a** and **14b** with the calculated ECD spectra of (*S*,*S_S_*)-**14** and (*R*,*S_S_*)-**14** in ACN; Figure S6: Comparison of the experimental ECD spectra of **15a** and **15b** with the calculated ECD spectra of (*S*)-**15** and (*R*)-**15** in ACN; Figure S7: The inhibitory activities of 4a/4b against kinase C-KIT(D816V); NMR and HRMS of intermediates and **4a**/**4b**. All authors have read and agreed to the published version of the manuscript.

## References

[1] Krupp F., Frey W., Richert C. (2020). Absolute configuration of small molecules by co-crystallization. Angew. Chem. Int. Ed..

[2] Oyama K.I., Kondo T., Shimizu T., Yoshida K. (2020). Determination of absolute configuration of photo-degraded catechinopyranocyanidin A by modified Mosher’s method. Chirality.

[3] Mandi A., Kurtan T. (2019). Applications of OR/ECD/VCD to the structure elucidation of natural products. Nat. Prod. Rep..

[4] Superchi S., Scafato P., Gorecki M., Pescitelli G. (2018). Absolute configuration determination by quantum mechanical calculation of chiroptical spectra: Basics and applications to fungal metabolites. Curr. Med. Chem..

[5] Wang F., Vasilyev V., Clayton A.H.A. (2022). Optical spectra and conformation pool of tyrosine kinase inhibitor PD153035 using a robust quantum mechanical conformation search. New J. Chem..

[6] Batista A.N.L., Valverde A.L., Nafie L.A., Batista J.M. (2024). Stereochemistry of natural products from vibrational circular dichroism. Chem. Commun..

[7] Parsons S. (2017). Determination of absolute configuration using X-ray diffraction. Tetrahedron Asymmetr.

[8] Cabral T.L.G., Poggetto G.D., da Silva J.P.B., Nilsson M., Tormena C.F. (2025). Determining the absolute configuration of small molecules by diffusion NMR experiments. Angew. Chem. Int. Ed..

[9] Yang B.B., Gao F., Yang Y.D., Wang R., Li X., Li L. (2023). Stereochemistry of chiral 2-substituted chromanes: Twist of the dihydropyran ring and specific optical rotation. Molecules.

[10] Padula D., Pescitelli G. (2018). How and how much molecular conformation affects electronic circular dichroism: The case of 1,1-diarylcarbinols. Molecules.

[11] Brocks D.R. (2006). Drug disposition in three dimensions: An update on stereoselectivity in pharmacokinetics. Biopharm. Drug Dispos..

[12] McVicker R.U., O’Boyle N.M. (2024). Chirality of new drug approvals (2013–2022): Trends and perspectives. J. Med. Chem..

[13] Hoy S.M. (2024). Seladelpar: First approval. Drugs.

[14] Dhillon S. (2024). Tovorafenib: First approval. Drugs.

[15] Markham A., Duggan S. (2019). Darolutamide: First approval. Drugs.

[16] Dhillon S. (2020). Avapritinib: First approval. Drugs.

[17] Wu C.P., Lusvarghi S., Wang J.C., Hsiao S.H., Huang Y.H., Hung T.H., Ambudkar S.V. (2019). Avapritinib: A selective inhibitor of KIT and PDGFRα that reverses ABCB1 and ABCG2-mediated multidrug resistance in cancer cell lines. Mol. Pharm..

[18] Gotlib J., Reiter A., Radia D.H., Deininger M.W., George T.I., Panse J., Vannucchi A.M., Platzbecker U., Alvarez-Twose I., Mital A. (2021). Efficacy and safety of avapritinib in advanced systemic mastocytosis: Interim analysis of the phase 2 PATHFINDER trial. Nat. Med..

[19] Heinrich M.C., Zhang X., Jones R.L., George S., Serrano C., Deng Y., Bauer S., Cai S., Wu X., Zhou Y. (2024). Clinical benefit of avapritinib in KIT-mutant gastrointestinal stromal tumors: A post hoc analysis of the phase I NAVIGATOR and phase I/II CS3007-001 studies. Clin. Cancer Res..

[20] Pardanani A., Reichard K., Tefferi A. (2024). Advanced systemic mastocytosis-Revised classification, new drugs and how we treat. Br. J. Haematol..

[21] Hodous B.L., Kim J.K., Wilson K.J., Wilson D., Zhang Y. (2017). Compositions useful for treating disorders related to kit. U.S. Patent.

[22] Betzenbichler G., Huber L., Kräh S., Morkos M.-L.K., Siegle A.F., Trapp O. (2022). Chiral stationary phases and applications in gas chromatography. Chirality.

[23] Liu H., Wu Z., Chen J., Wang J., Qiu H. (2023). Recent advances in chiral liquid chromatography stationary phases for pharmaceutical analysis. J. Chromatogr. A.

[24] Lipka E. (2022). Contribution of supercritical fluid chromatography to serially coupling columns for chiral and achiral separations. TrAC-Trends Anal. Chem..

[25] Liu H., Chen J., Chen M., Wang J., Qiu H. (2023). Recent development of chiral ionic liquids for enantioseparation in liquid chromatography and capillary electrophoresis: A review. Anal. Chim. Acta.

[26] Berova N., Bari L.D., Pescitelli G. (2007). Application of electronic circular dichroism in configurational and conformational analysis of organic compounds. Chem. Soc. Rev..

[27] Li L., Si Y.K. (2011). Study on the absolute configuration of levetiracetam via density functional theory calculations of electronic circular dichroism and optical rotatory dispersion. J. Pharmaceut Biomed..

[28] Keiderling T.A. (2018). Instrumentation for vibrational circular dichroism spectroscopy: Method comparison and newer developments. Molecules.

[29] Demarque D.P., Heinrich S., Schulz F., Merten C. (2020). Sensitivity of VCD spectroscopy for small structural and stereochemical changes of macrolide antibiotics. Chem. Commun..

[30] Gorecki M., Zullo V., Iuliano A., Pescitelli G. (2019). On the absolute stereochemistry of tolterodine: A circular dichroism study. Pharmaceuticals.

[31] Zhang J.Y., Yang B.B., Yang Y.D., Gao F., Liu W.Q., Li L. (2021). Correlations between the ECD spectra and absolute configuration of bridged-ring lactones: Revisiting Beecham’s rule. Org. Biomol. Chem..

[32] Giorgio E., Viglione R.G., Zanasi R., Rosini C. (2004). Ab initio calculation of optical rotatory dispersion (ORD) curves: A simple and reliable approach to the assignment of the molecular absolute configuration. J. Am. Chem. Soc..

[33] Lu J.M., Yang B.B., Li L. (2020). Specific optical rotation and absolute configuration of flexible molecules containing a 2-methylbutyl residue. Eur. J. Org. Chem..

[34] Byers L.A., Horn L., Ghandi J., Kloecker G., Owonikoko T., Waqar S.N., Krzakowski M., Cardnell R.J., Fujimoto J., Taverna P. (2017). A phase 2, open-label, multi-center study of amuvatinib in combination with platinum etoposide chemotherapy in platinum-refractory small cell lung cancer patients. Oncotarget.

[35] Teuber A., Schulz T., Fletcher B.S., Gontla R., Mühlenberg T., Zischinsky M.-L., Niggenaber J., Weisner J., Kleinbölting S.B., Lategahn J. (2024). Avapritinib-based SAR studies unveil a binding pocket in KIT and PDGFRA. Nat. Commun..

[36] MOE2009.10, Chemical Computing Group Inc.. https://www.chemcomp.com/en/Products.htm.

[37] Frisch M.J., Trucks G.W., Schlegel H.B., Scuseria G.E., Robb M.A., Cheeseman J.R., Scalmani G., Barone V., Mennucci B., Petersson G.A. (2016). Gaussian 16, Revision, B.01.

[38] Bruhn T., Schaumlöffel A.N.U., Hemberger Y., Bringmann G. (2013). SpecDis: Quantifying the comparison of calculated and experimental electronic circular dichroism spectra. Chirality.

[39] (2017). SpecDis.

