# Supplementary material for: Absolute Configuration and Chiroptical Properties of Flexible Drug Avapritinib
Source: Pharmaceuticals (Basel). 2025 Jun 2;18(6):833. doi: 10.3390/ph18060833 (PMC12196291; doi:10.3390/ph18060833)

Supplementary Materials

Absolute Configuration and Chiroptical Properties of Flexible Drug Avapritinib

Ya-Dong Yang†, Chen Zhao†, Liang-Peng Li, Yi-Xin Lv, Bei-Bei Yang, Xin Li, Ru Wang and Li Li*

State Key Laboratory of Digestive Health, Beijing Key Laboratory of Active Substances Discovery and Druggability Evaluation, Institute of Materia Medica, Chinese Academy of Medical Sciences & Peking Union Medical College, Beijing 100050, China

Contents

Chiral HPLC conditions of compounds **4** and **15** S1

Conformational grouping of (*S*)-**4** S2

Experimental ECD spectra of **4a** and **4b** in methanol and water S6

Boltzmann distribution of (*S*)-**4** obtained using three calculation methods S6

Calculated ECD spectra of (*S*)-**5** using three calculation methods S7

ORD curves of **15a**/**15b** S7

Experimental and calculated ECD of **14a/b** and hydrochloride salt of **15a/b** S8

Inhibitory activities of **4** against kinase C-KIT(D816V) S9

^{1}H NMR and ESI-HRMS of **10-15/4a/4b** S10

Table S1. Chiral HPLC conditions of compounds **4** and **15**

Compound **4**	
Column	CHIRALPAK IG
Column size	0.46 cm I.D. × 15 cm L
Injection	5.0 μL
Mobile phase	CH_2Cl_2/CH_3OH DEA0.1% = 60/40 (V/V)
Flow rate	1.0 mL/min
Wave length	254 nm
Temperature	25 °C
Compound **15**	
Column	CHIRALPAK ID
Column size	0.46 cm I.D. × 15 cm L
Injection	1.0 μL
Mobile phase	CH_3OH 0.1%DEA = 100%
Flow rate	1.0 mL/min
Wave length	254 nm
Temperature	25 °C

Table S2. Conformational grouping of (*S*)-**4**

Conformer		Cis[a]		Trans	
		Methyl coplanar[b]	Methyl non-coplanar	Methyl coplanar	Methyl non-coplanar
E[c]	Far[d]	Group 1 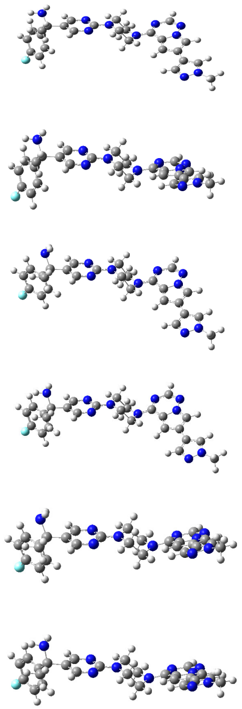	Group 3 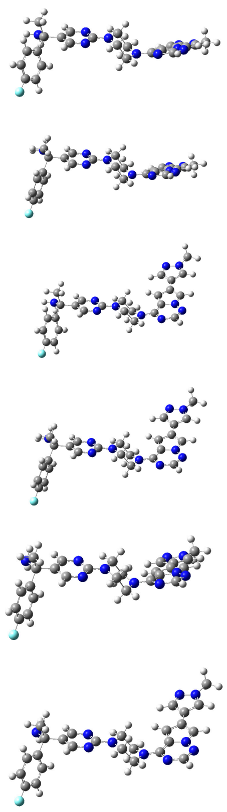	Group 9 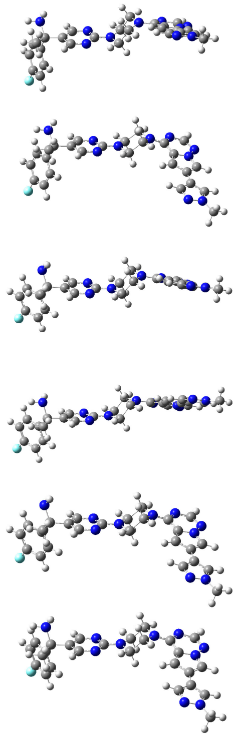	Group 11 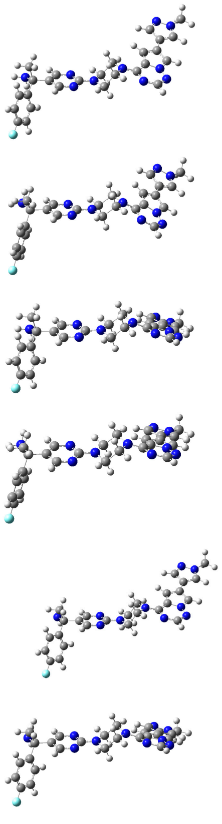

		Group 2	Group 4	Group 10	Group 12
	near	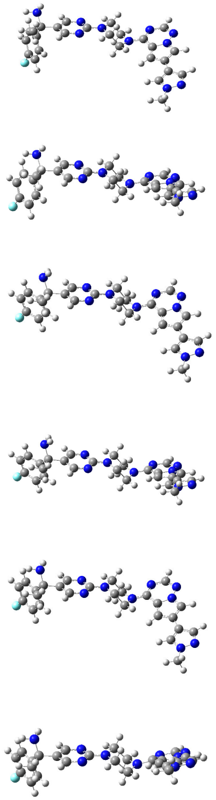	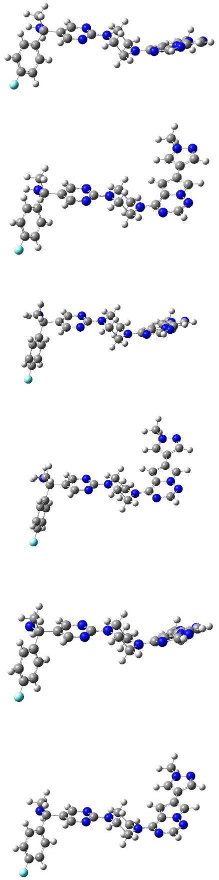	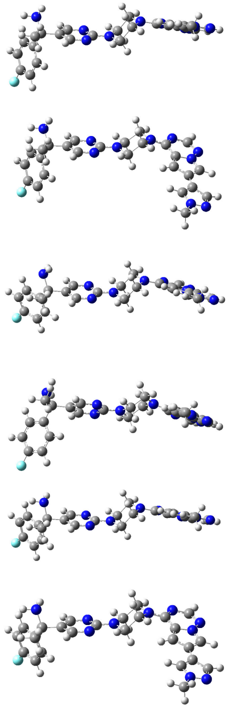	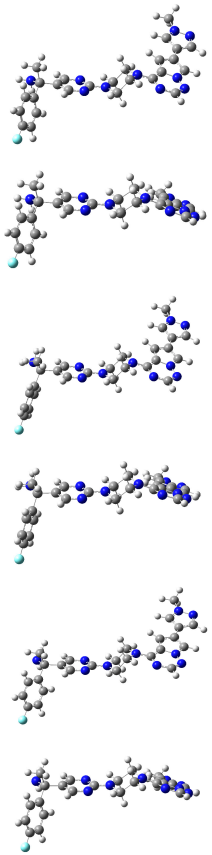

Z	far	Group 5 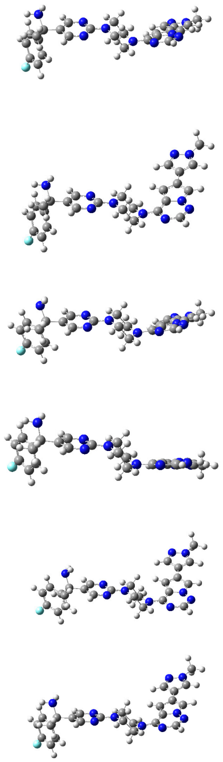	Group 7 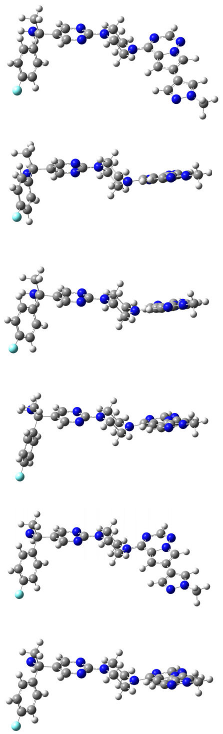	Group 13 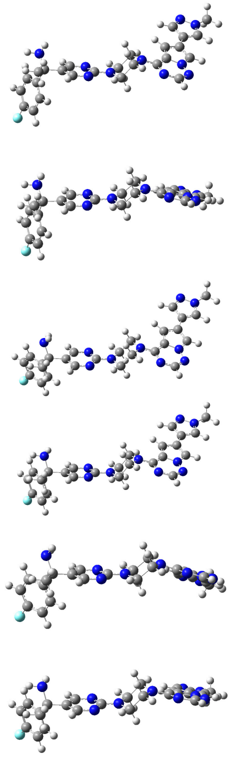	Group 15 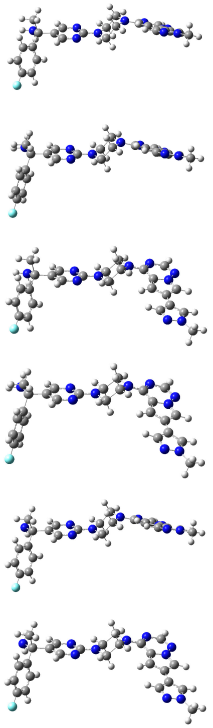

	near	Group 6 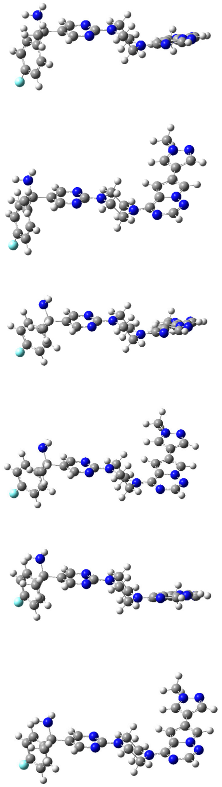	Group 8 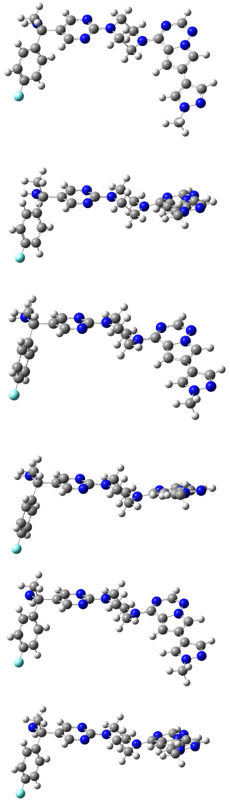	Group 14 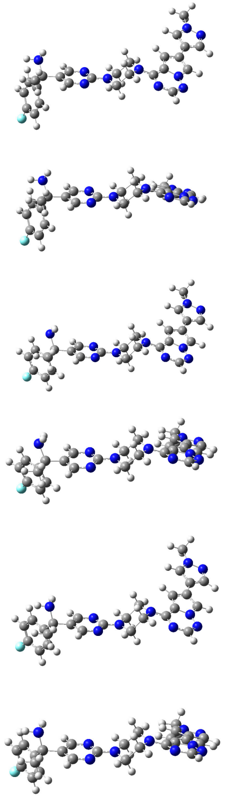	Group 16 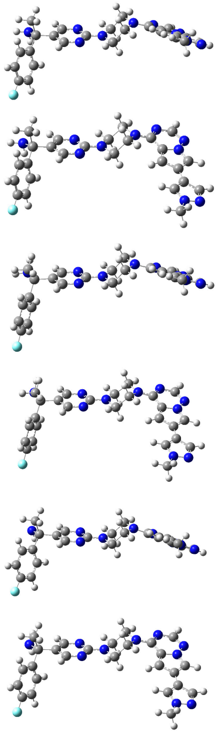

a). Cis/trans represents the fragments I and III stay on the different side or same side of fragment II;

b). Methyl coplanar/ Methyl non-coplanar represents the dihedral angle C27-C19-C20-C25 value is close to 180° or in the range of 50-100°;

c). E/Z represents the fluorine atom on the fragment I and pyrazole ring of fragment III toward the different direction and same directions;

d). Far/near represents the terminal methyl may close to the core or far from the core.

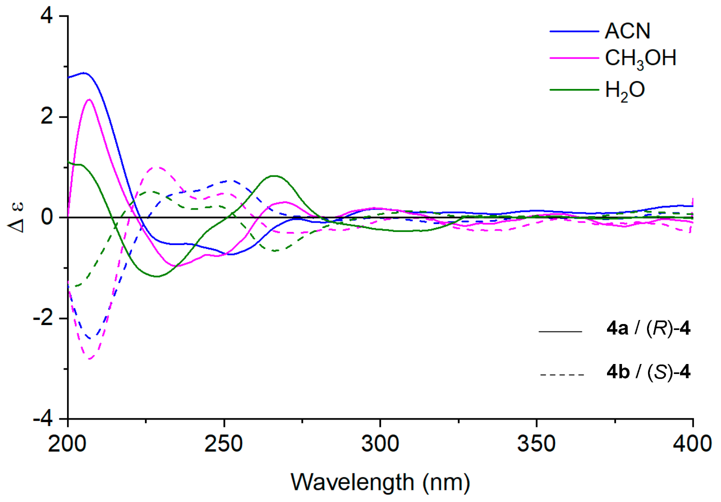

Figure S1. Experimental ECD spectra of **4a** and **4b** in methanol and water.

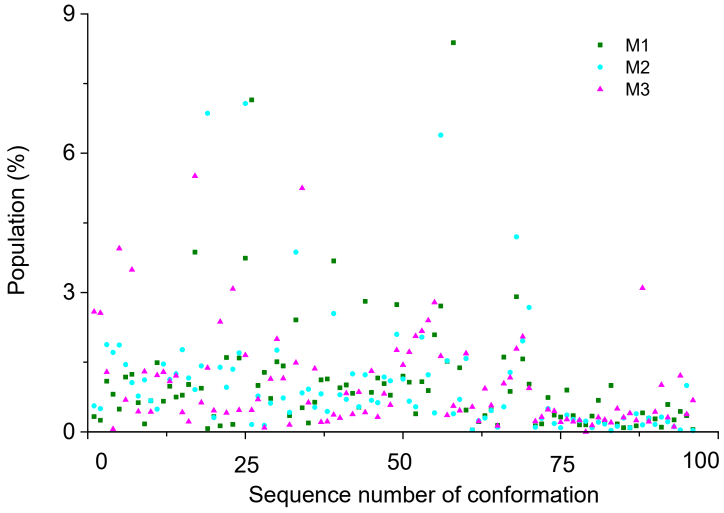

Figure S2. Boltzmann distribution of (*S*)-**4** obtained using three calculation methods.
M1: SMD/ACN/B3LYP/6-31G(d,p); M2: SMD/CH_2Cl_2/B3LYP/6-31G(d,p); M3: SMD/CH_2Cl_2/B3LYP/6-31++G(d,p).

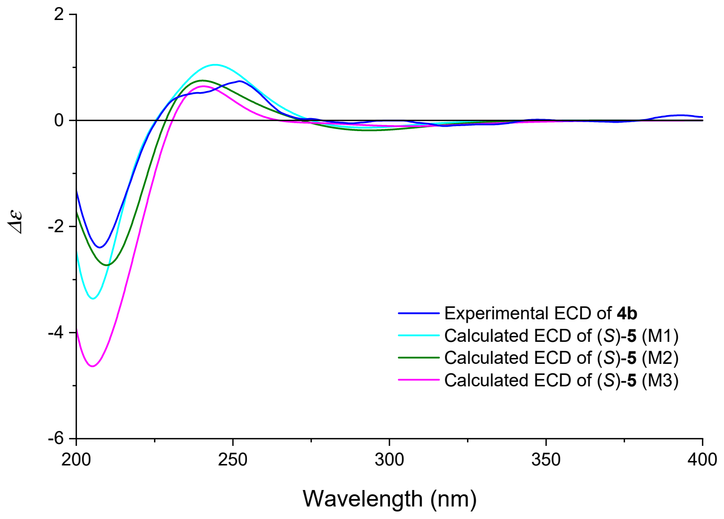

Figure S3. Comparison of the experimental ECD spectrum of **4b** with the calculated ECD spectra of (*S*)-**5** in ACN using different methods.

M1: SMD/ACN/B3LYP/6-31G(d,p)//B3LYP/6-31G(d,p), M2: SMD/ACN/Cam-B3LYP/6-311G (d,p)//Cam-B3LYP/6-311G(d,p), M3: SMD/ACN/ωB97XD/TZVP//ωB97XD/TZVP.

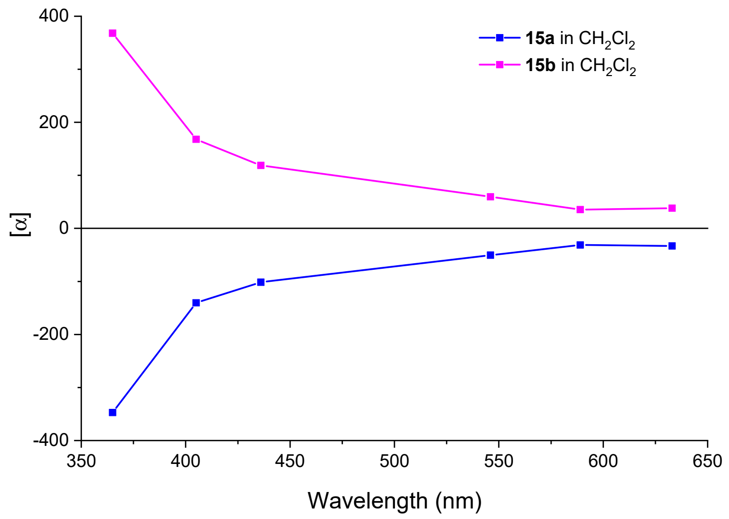

Figure S4. ORD curves of **15a** and **15b** in CH_2Cl_2.

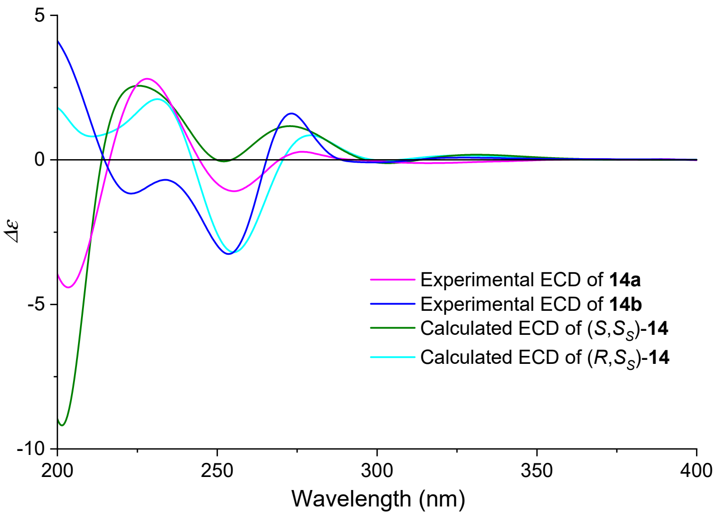

Figure S5. Comparison of the experimental ECD spectra of **14a** and **14b** with the calculated ECD spectra of (*S*,S_S)-**14** and (*R*,S_S)-**14** in ACN.

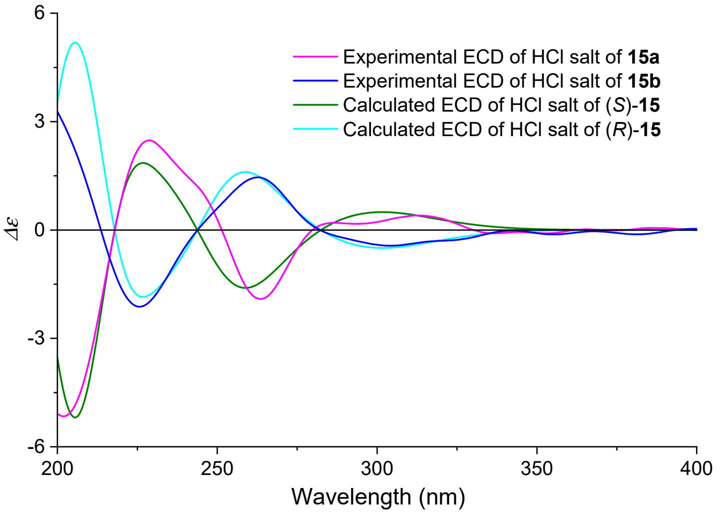

Figure S6. Comparison of the experimental ECD spectra of **15a** and **15b** with the calculated ECD spectra of (*S*)-**15** and (*R*)-**15** in ACN.

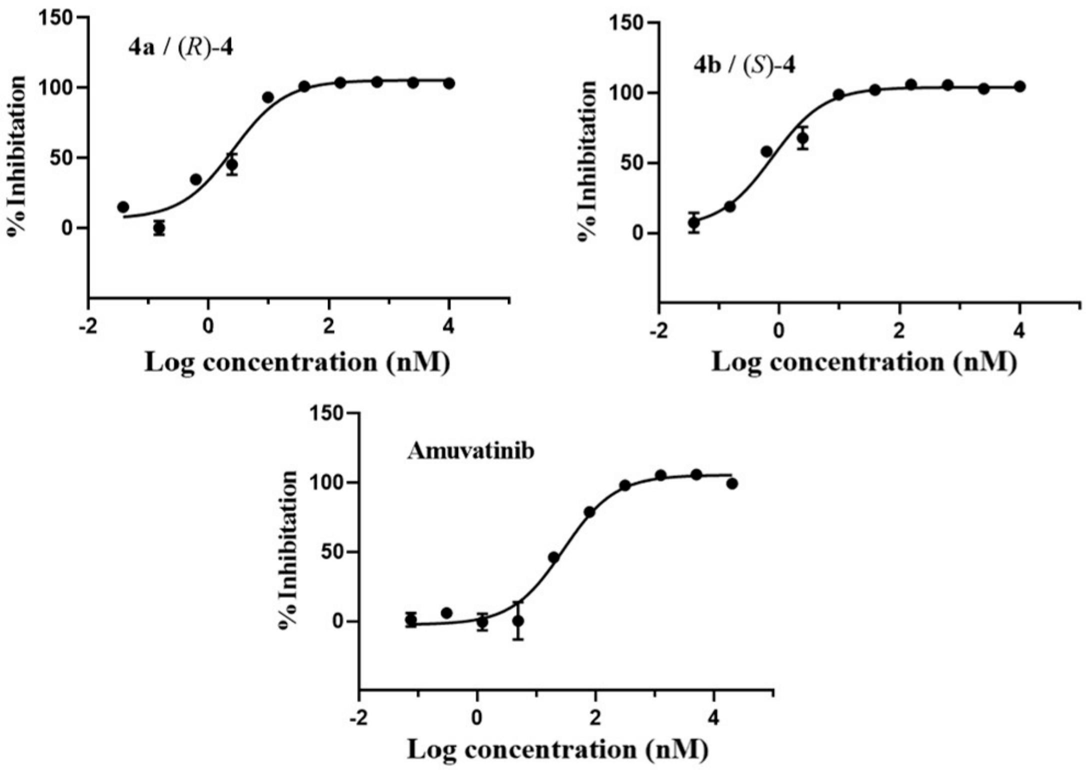

Figure S7. The inhibitory activities of **4a**/**4b** against kinase C-KIT(D816V).

^{1}H NMR spectra and ESI-HRMS of **10**

(1) 400 MHz ^{1}HNMR spectrum of **10**

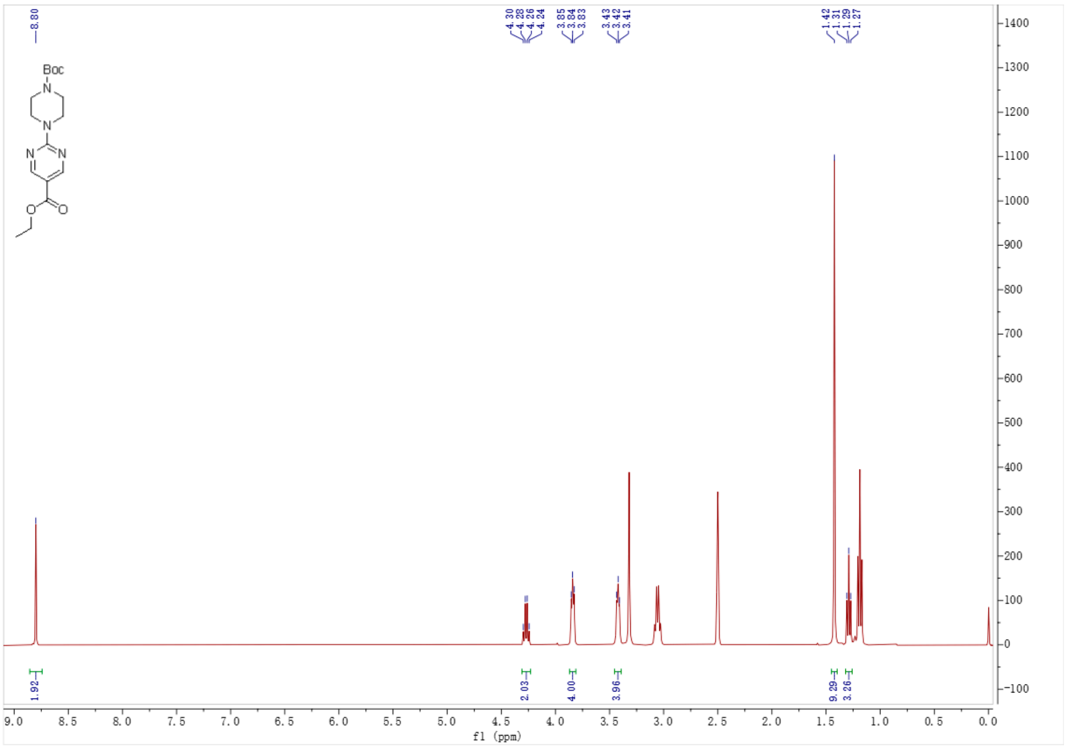

(2) ESI-HRMS spectrum of **10**

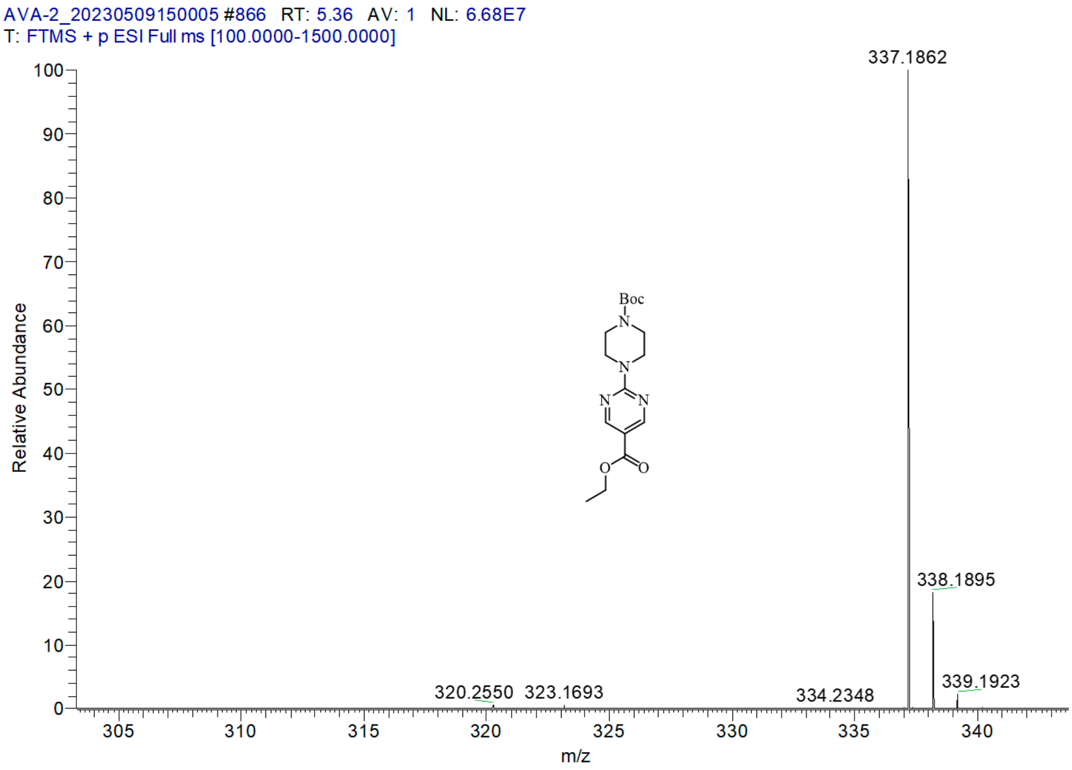

^{1}H NMR spectra and MS of 11

(1) 400 MHz ^{1}HNMR spectrum of **11**

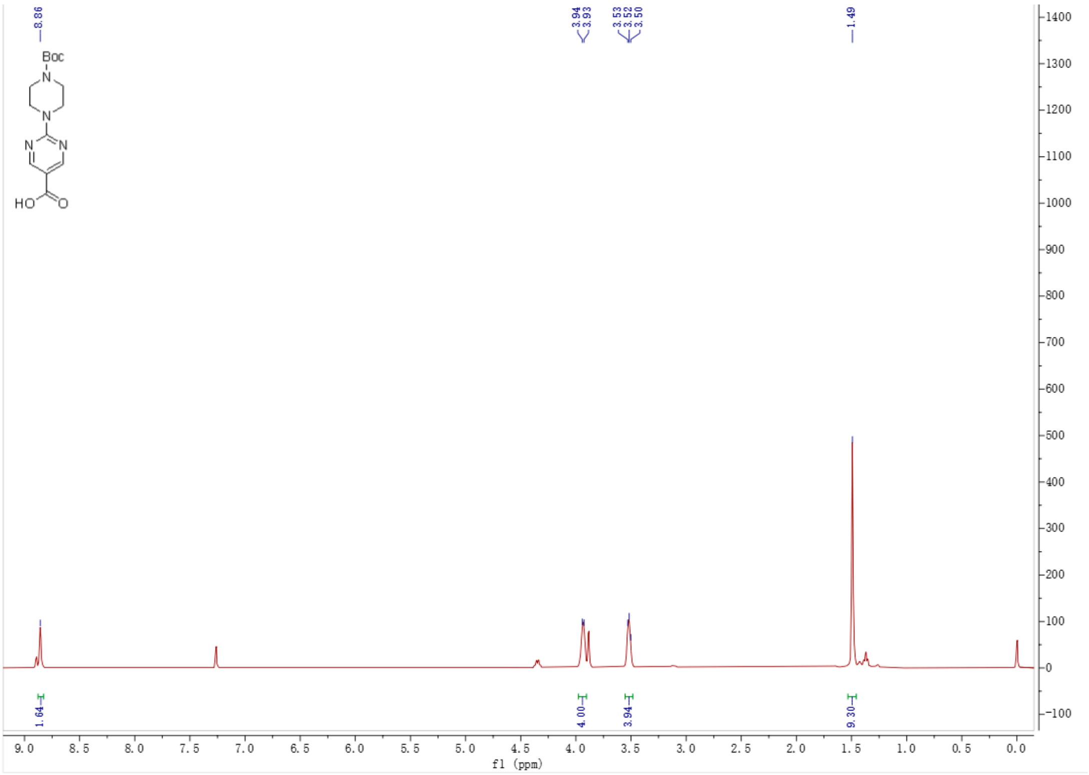

(2) ESI-HRMS spectrum of **11**

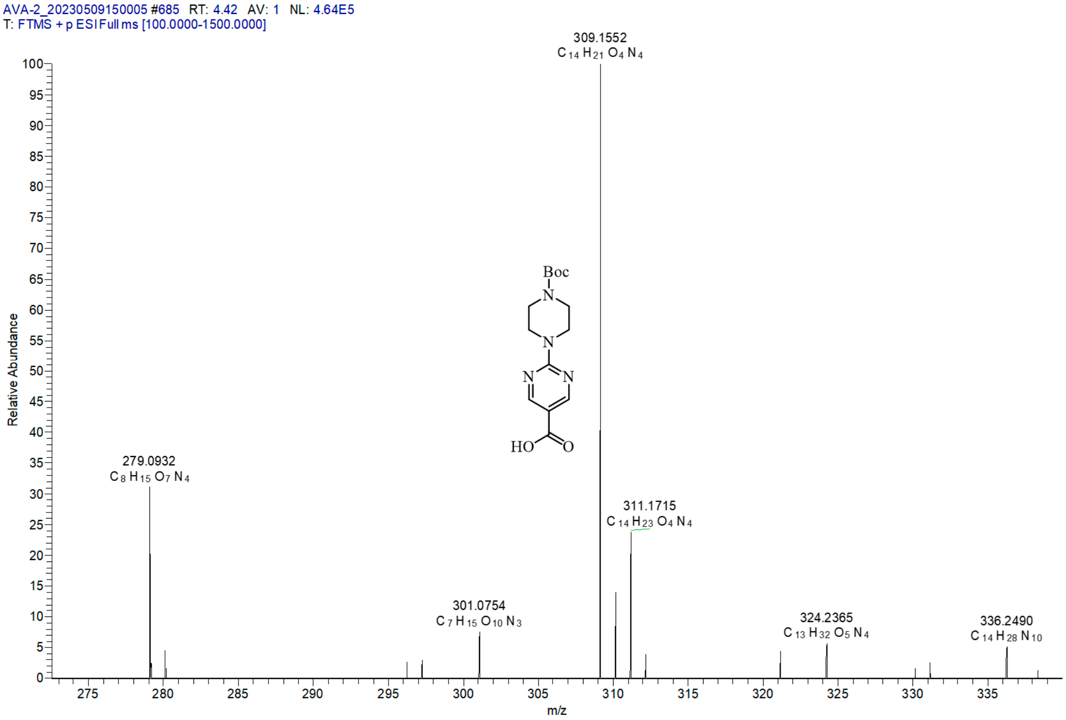

^{1}H NMR spectra and MS of 12

(1) 400 MHz ^{1}HNMR spectrum of **12**

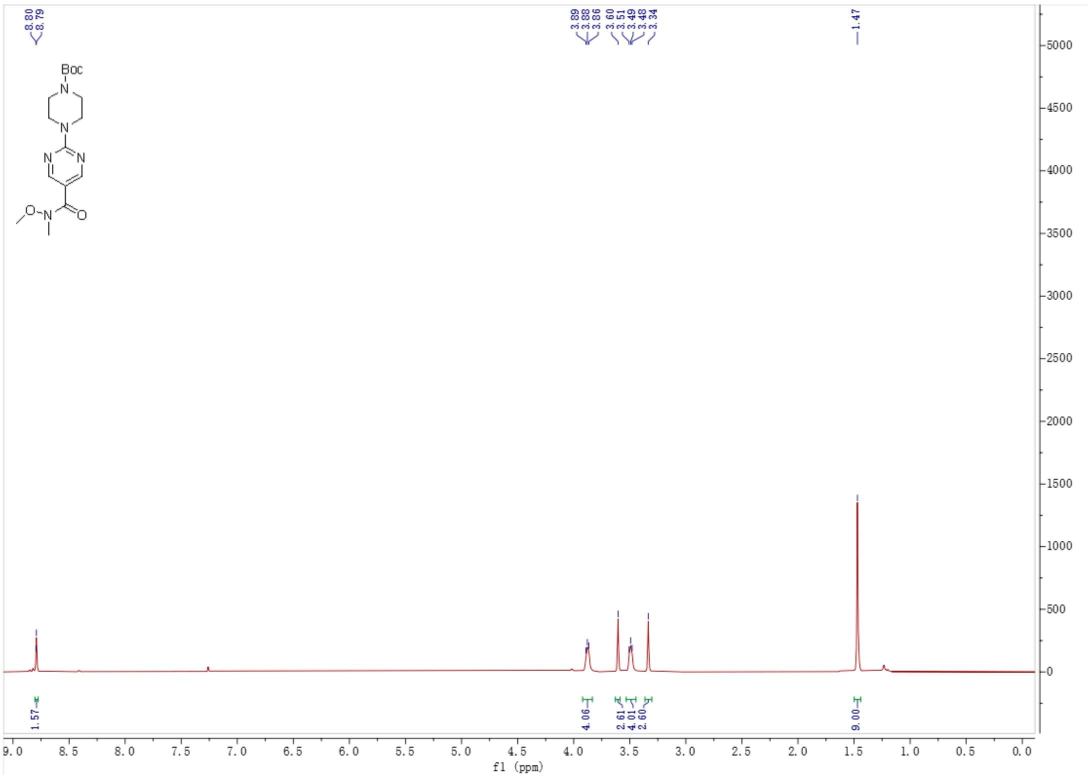

(2) ESI-HRMS spectrum of **12**

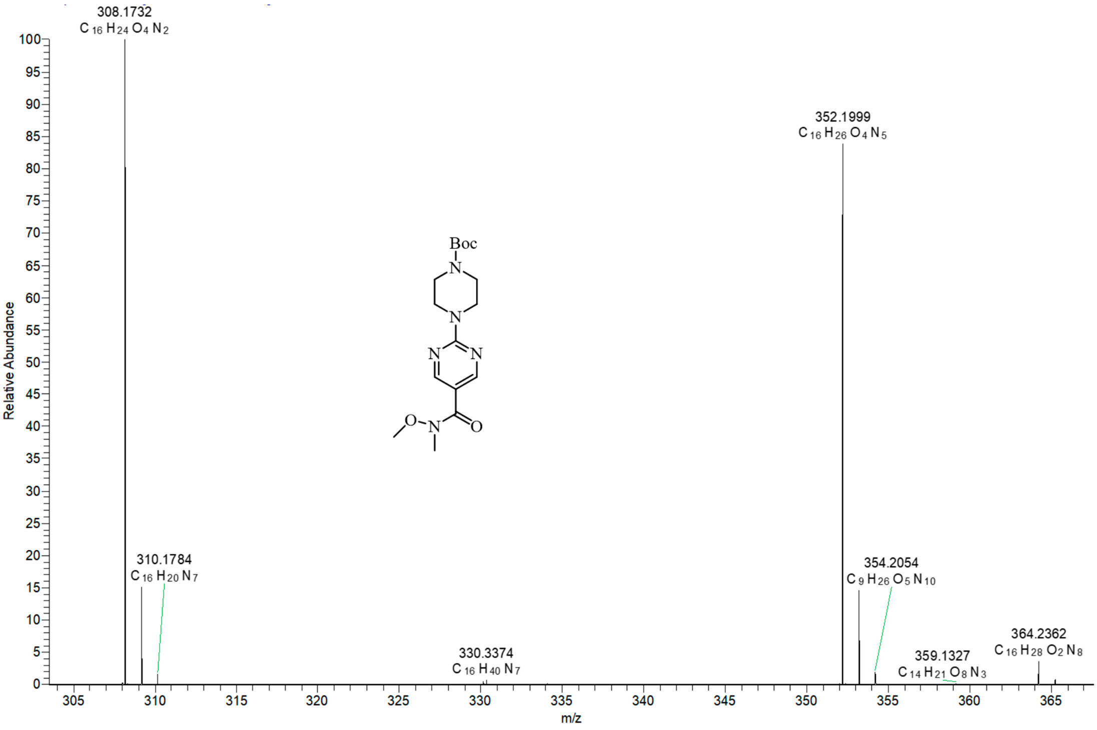

^{1}H NMR spectra and MS of 13

(1) 400 MHz ^{1}HNMR spectrum of **13**

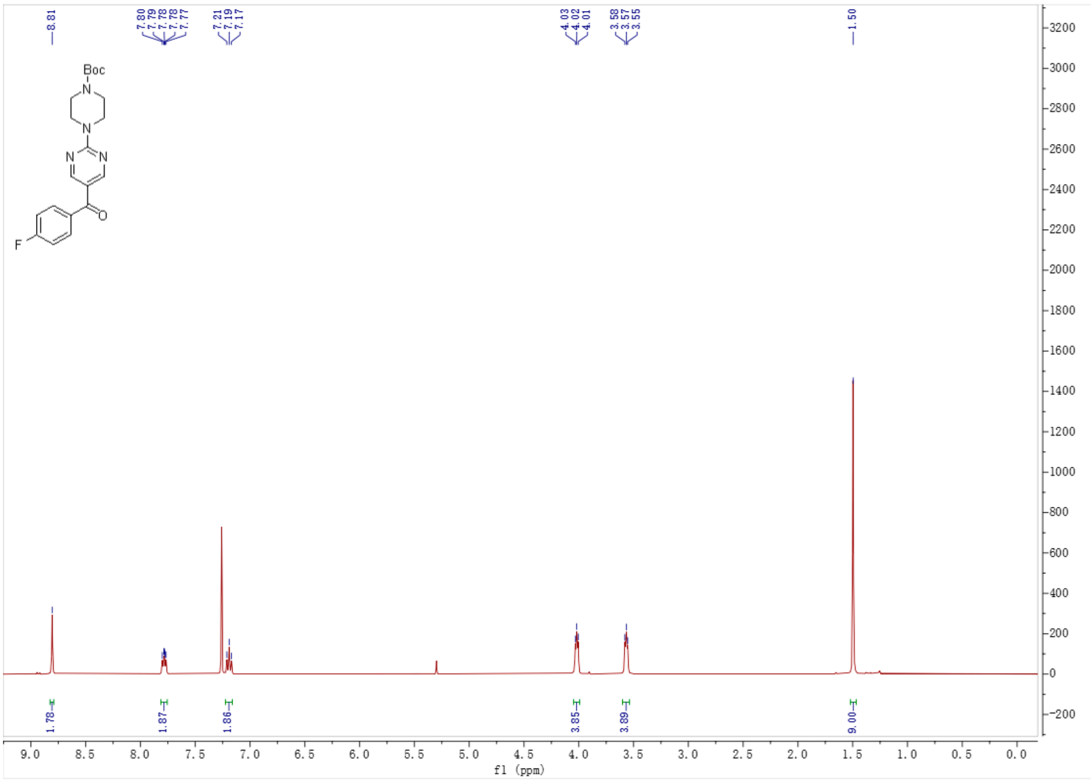

(2) ESI-HRMS spectrum of **13**

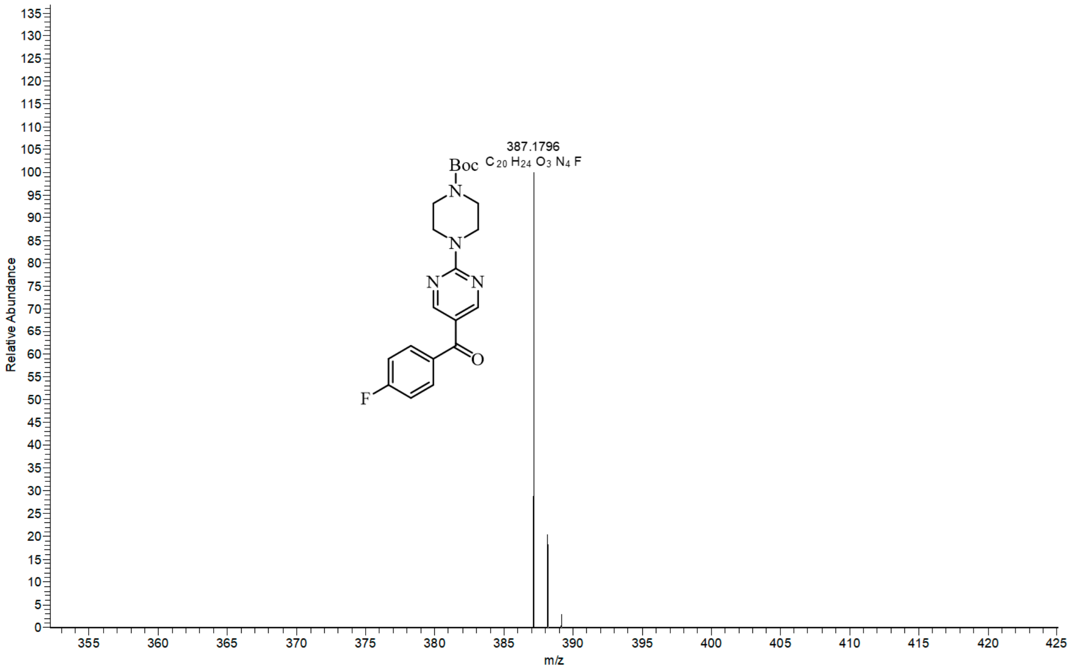

^{1}H NMR spectra and MS of **14**

(1) 400 MHz ^{1}HNMR spectrum of **14**

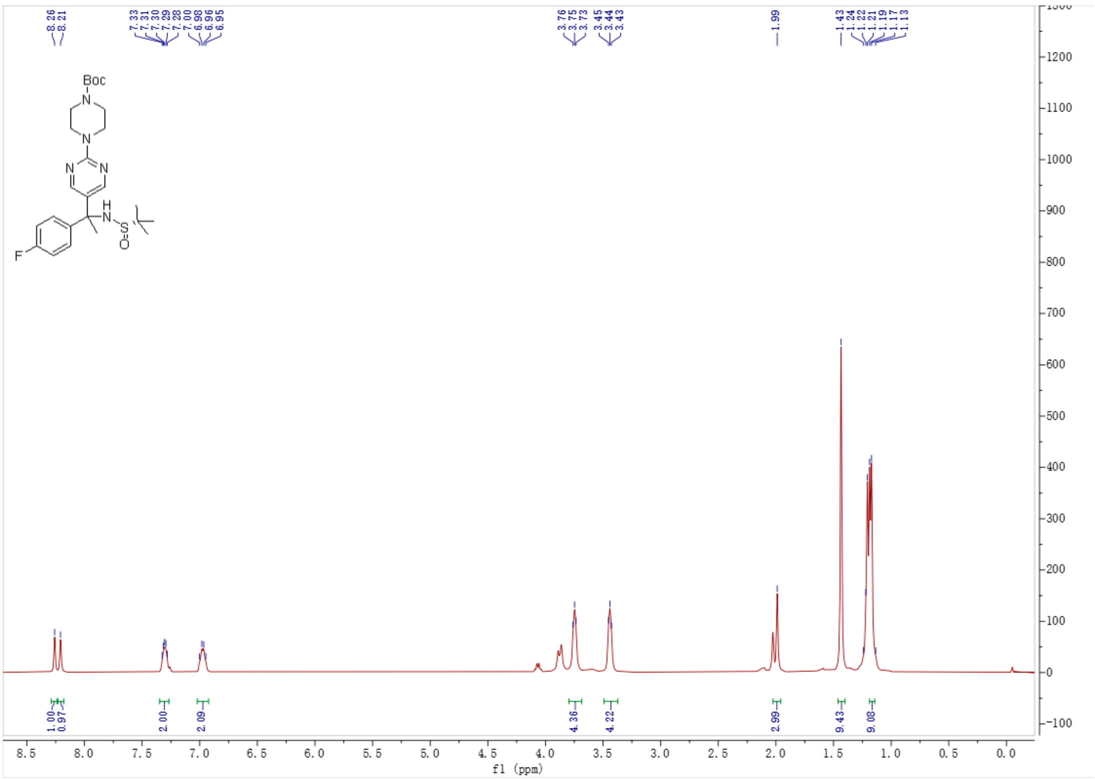

(2) ESI-HRMS spectrum of **14**

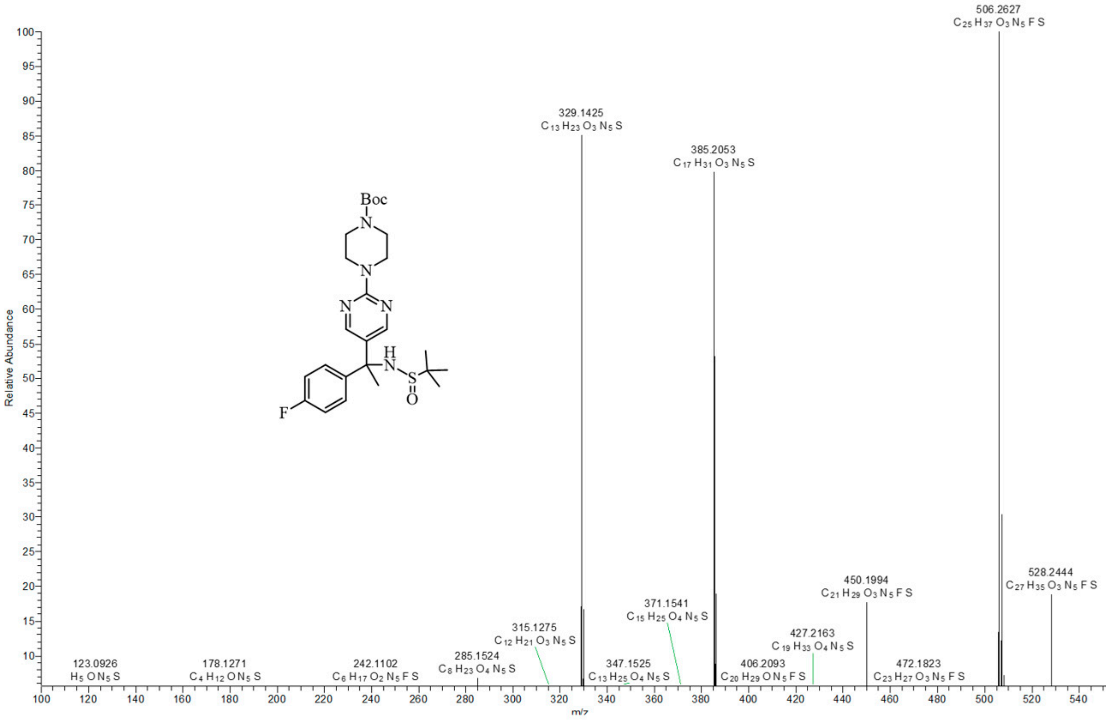

^{1}H NMR spectra and MS of 15

(1) 400 MHz ^{1}HNMR spectrum of **15**

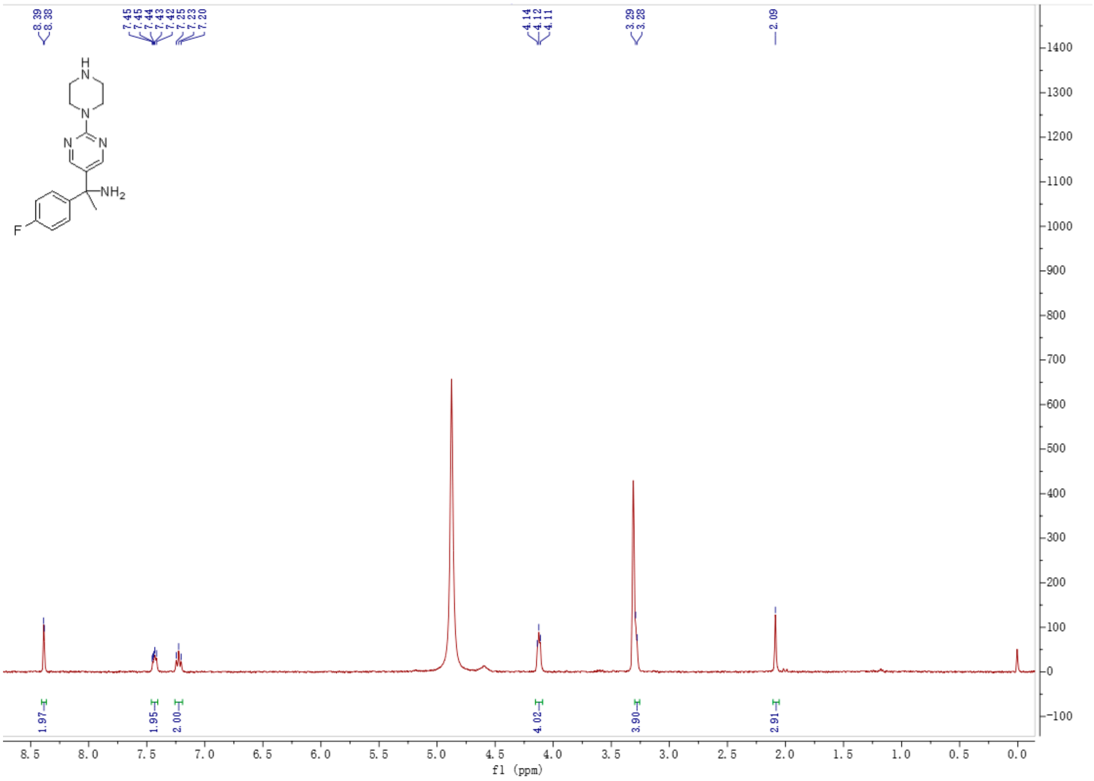

(2) ESI-HRMS spectrum of **15**

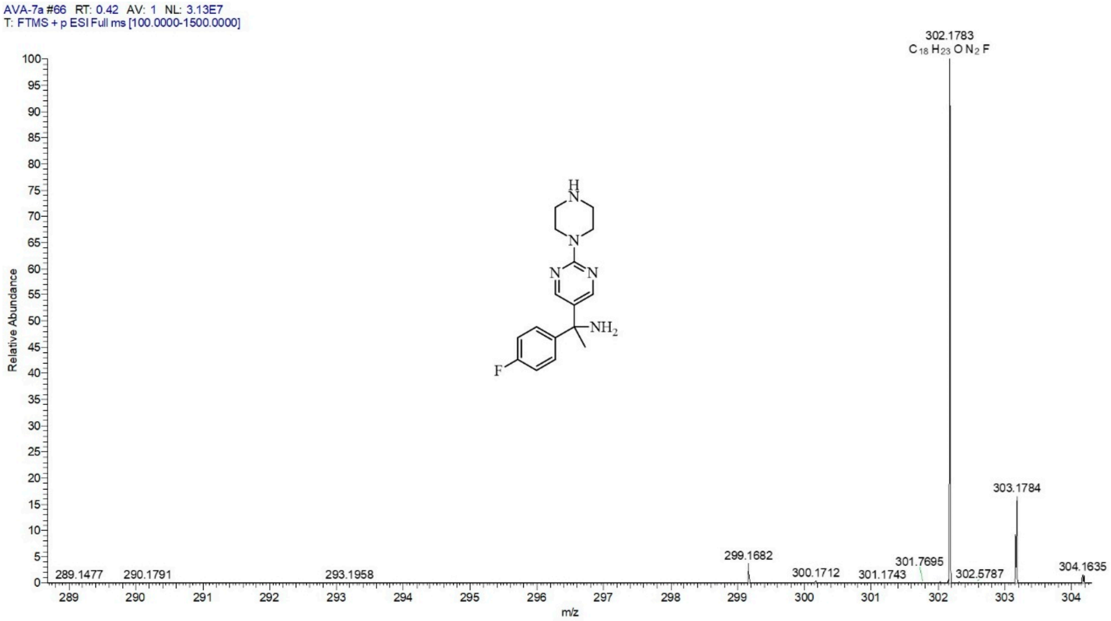

(3) 500 MHz ^{1}HNMR spectrum of **15a**

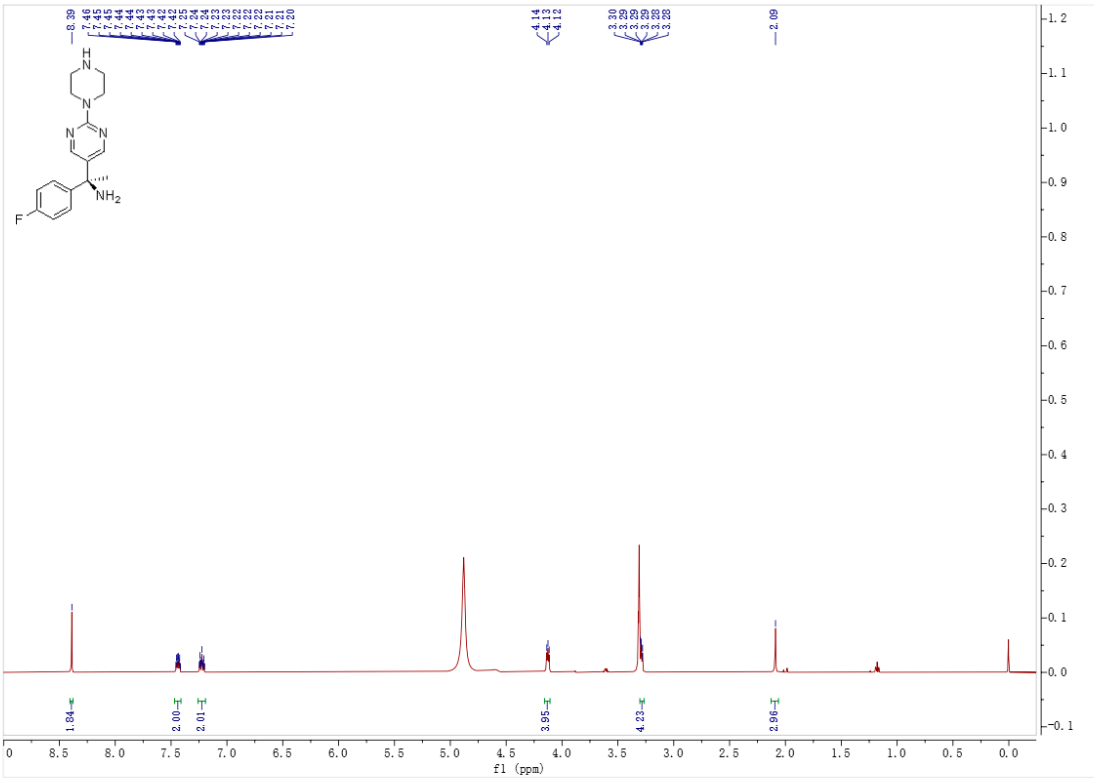

(4) 500 MHz ^{1}HNMR spectrum of **15b**

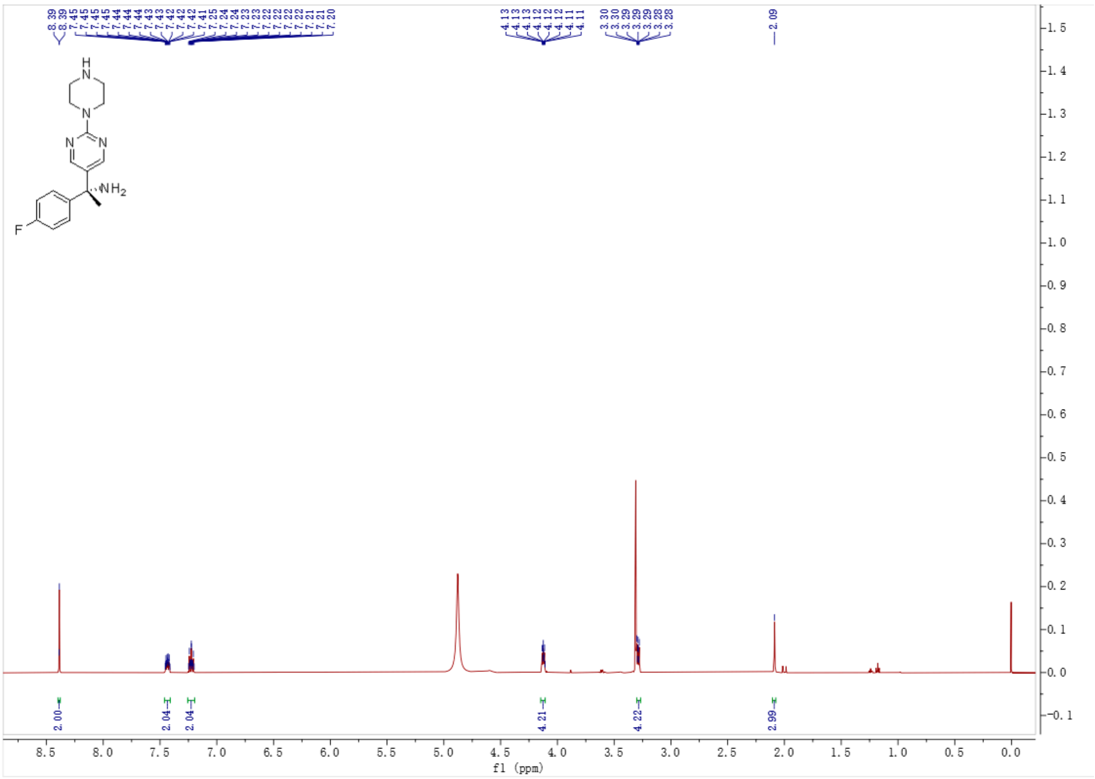

^{1}H NMR spectra and MS of 4

(1) 400 MHz ^{1}HNMR spectrum of **4a**

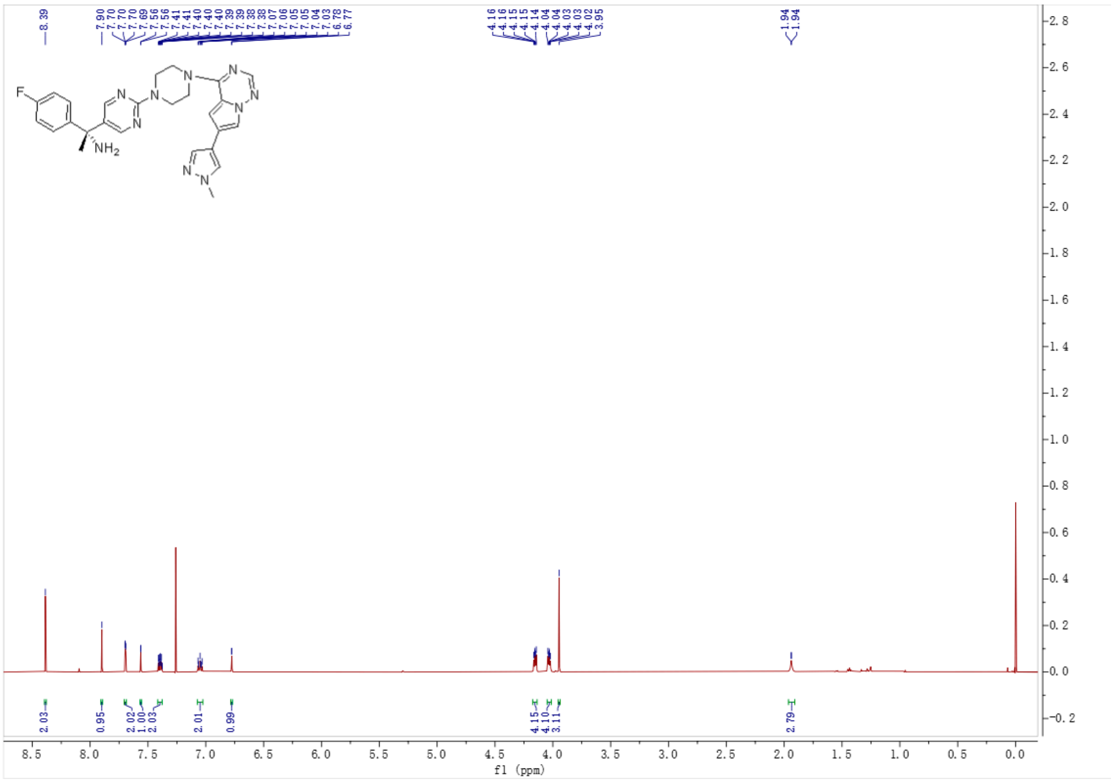

(2) ESI-HRMS spectrum of **4a**

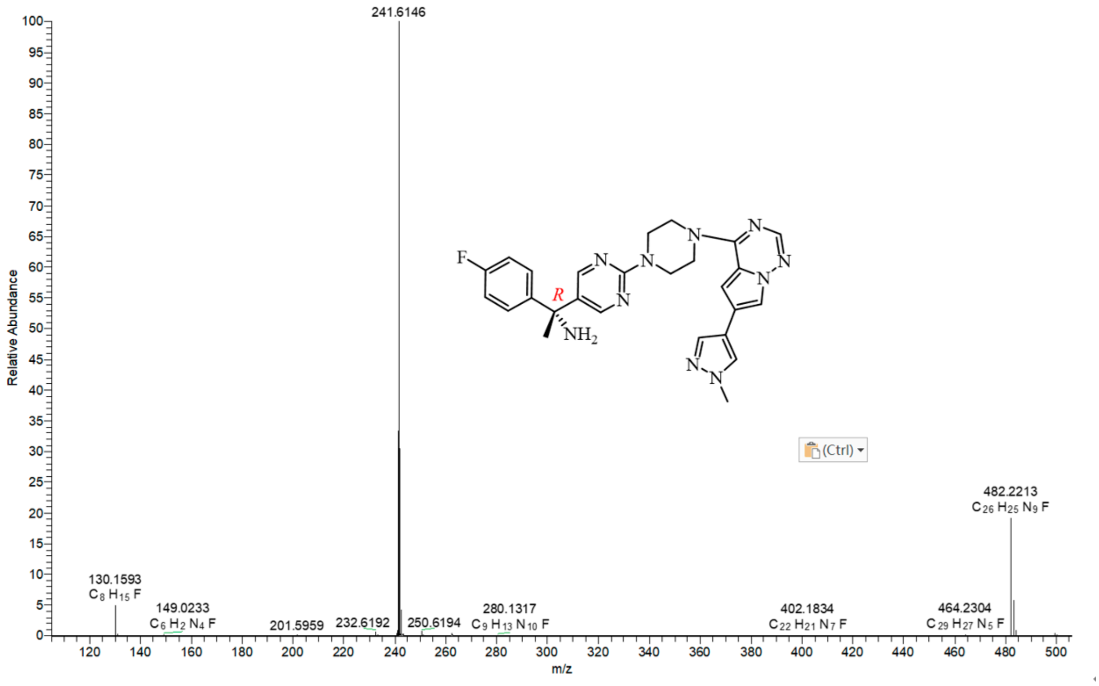

(3) 400 MHz ^{1}HNMR spectrum of **4b**

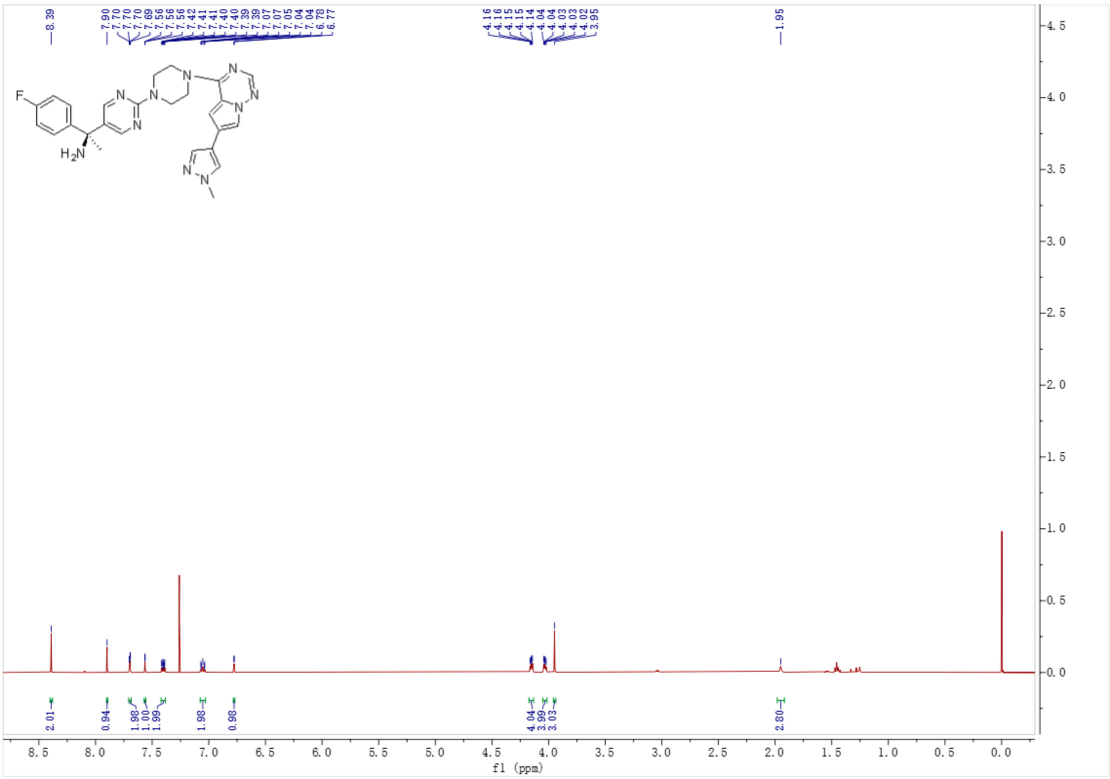

(4) ESI-HRMS spectrum of **4b**

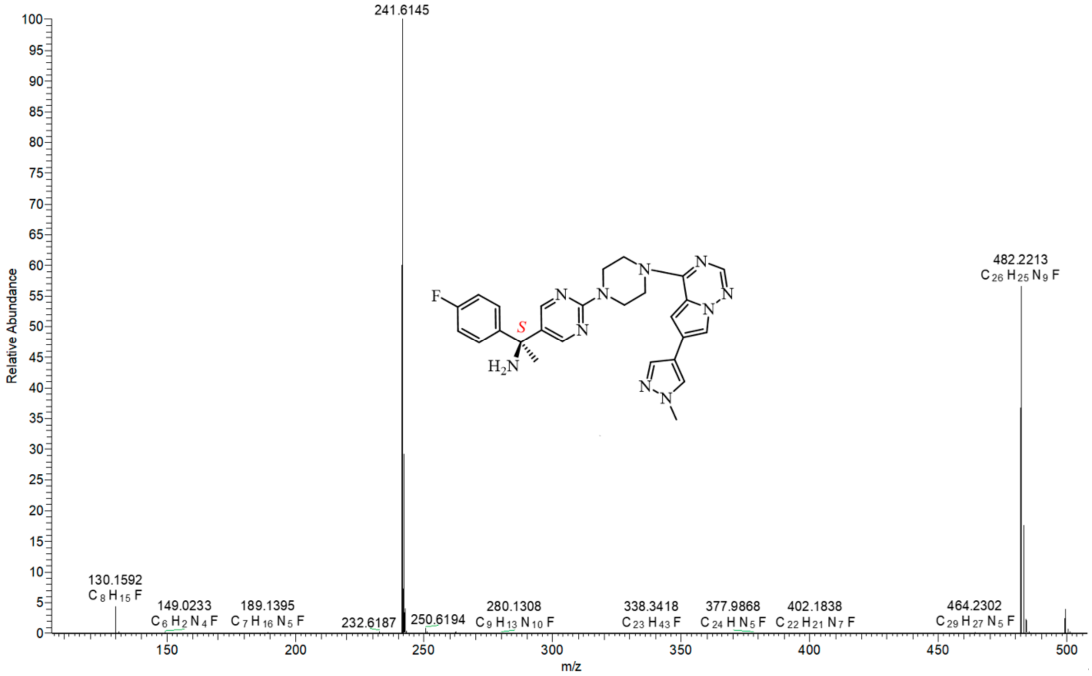

Supplement: Supplementary file 1 [file pharmaceuticals-18-00833-s001.zip › pharmaceuticals-3638167-supplementary.pdf]
